# Sustainable production of photosynthetic isobutanol and 3-methyl-1-butanol in the cyanobacterium *Synechocystis* sp. PCC 6803

**DOI:** 10.1186/s13068-023-02385-1

**Published:** 2023-09-09

**Authors:** Hao Xie, Jarl Kjellström, Peter Lindblad

**Affiliations:** Microbial Chemistry, Department of Chemistry-Ångström Laboratory, Uppsala University, Box 523, 75120 Uppsala, Sweden

**Keywords:** 2-Keto acid pathway, Isobutanol, 3-Methyl-1-butanol, Cyanobacteria, *Synechocystis* sp. PCC 6803, Metabolic engineering, Solar fuel

## Abstract

**Background:**

Cyanobacteria are emerging as green cell factories for sustainable biofuel and chemical production, due to their photosynthetic ability to use solar energy, carbon dioxide and water in a direct process. The model cyanobacterial strain *Synechocystis* sp. PCC 6803 has been engineered for the isobutanol and 3-methyl-1-butanol production by introducing a synthetic 2-keto acid pathway. However, the achieved productions still remained low. In the present study, diverse metabolic engineering strategies were implemented in *Synechocystis* sp. PCC 6803 for further enhanced photosynthetic isobutanol and 3-methyl-1-butanol production.

**Results:**

Long-term cultivation was performed on two selected strains resulting in maximum cumulative isobutanol and 3-methyl-1-butanol titers of 1247 mg L^−1^ and 389 mg L^−1^, on day 58 and day 48, respectively. Novel *Synechocystis* strain integrated with a native 2-keto acid pathway was generated and showed a production of 98 mg isobutanol L^−1^ in short-term screening experiments. Enhanced isobutanol and 3-methyl-1-butanol production was observed when increasing the *kivd*^*S286T*^ copy number from three to four. Isobutanol and 3-methyl-1-butanol production was effectively improved when overexpressing selected genes of the central carbon metabolism. Identified genes are potential metabolic engineering targets to further enhance productivity of pyruvate-derived bioproducts in cyanobacteria.

**Conclusions:**

Enhanced isobutanol and 3-methyl-1-butanol production was successfully achieved in *Synechocystis* sp. PCC 6803 strains through diverse metabolic engineering strategies. The maximum cumulative isobutanol and 3-methyl-1-butanol titers, 1247 mg L^−1^ and 389 mg L^−1^, respectively, represent the current highest value reported. The significantly enhanced isobutanol and 3-methyl-1-butanol production in this study further pave the way for an industrial application of photosynthetic cyanobacteria-based biofuel and chemical synthesis from CO_2_.

**Supplementary Information:**

The online version contains supplementary material available at 10.1186/s13068-023-02385-1.

## Introduction

In 2020, fossil resources supplied approximately 81% of total energy, whereas renewable resources accounted for approximately 15% of the total energy [1]. By 2050, the global energy demand is projected to increase by 47% [1]. In face of the rapid climate change and increasing energy demand, it is urgent to gradually replace traditional fossil resources with renewable energy, such as biofuels produced, e.g., by metabolically engineered microorganisms feeding on renewable carbon sources [2, 3]. Being generated from renewable resources, biofuels are cleaner energy as they release lower amounts of sulfates and black carbon particulates after burning [4]. Currently, bioethanol, mainly produced from biomass fermentation using sugarcane and corn as feedstocks, is the main biofuel used as gasoline additive. However, the energy density of ethanol is only 66% of gasoline, making it less favorable as gasoline additive compared to advanced alcohols. Isobutanol (IB), a four-carbon advanced alcohol, is recognized as a superior substitution as drop-in fuel, due to the following advantages: higher energy density, lower water solubility, lower vapor pressure and lower hygroscopicity compared to ethanol [5]. The boiling point and melting point of IB are + 108 °C and -108 °C. Moreover, IB and water form a heterogeneous azeotrope and protocols for separation by distillation are available [6].

**Fig. 1:**
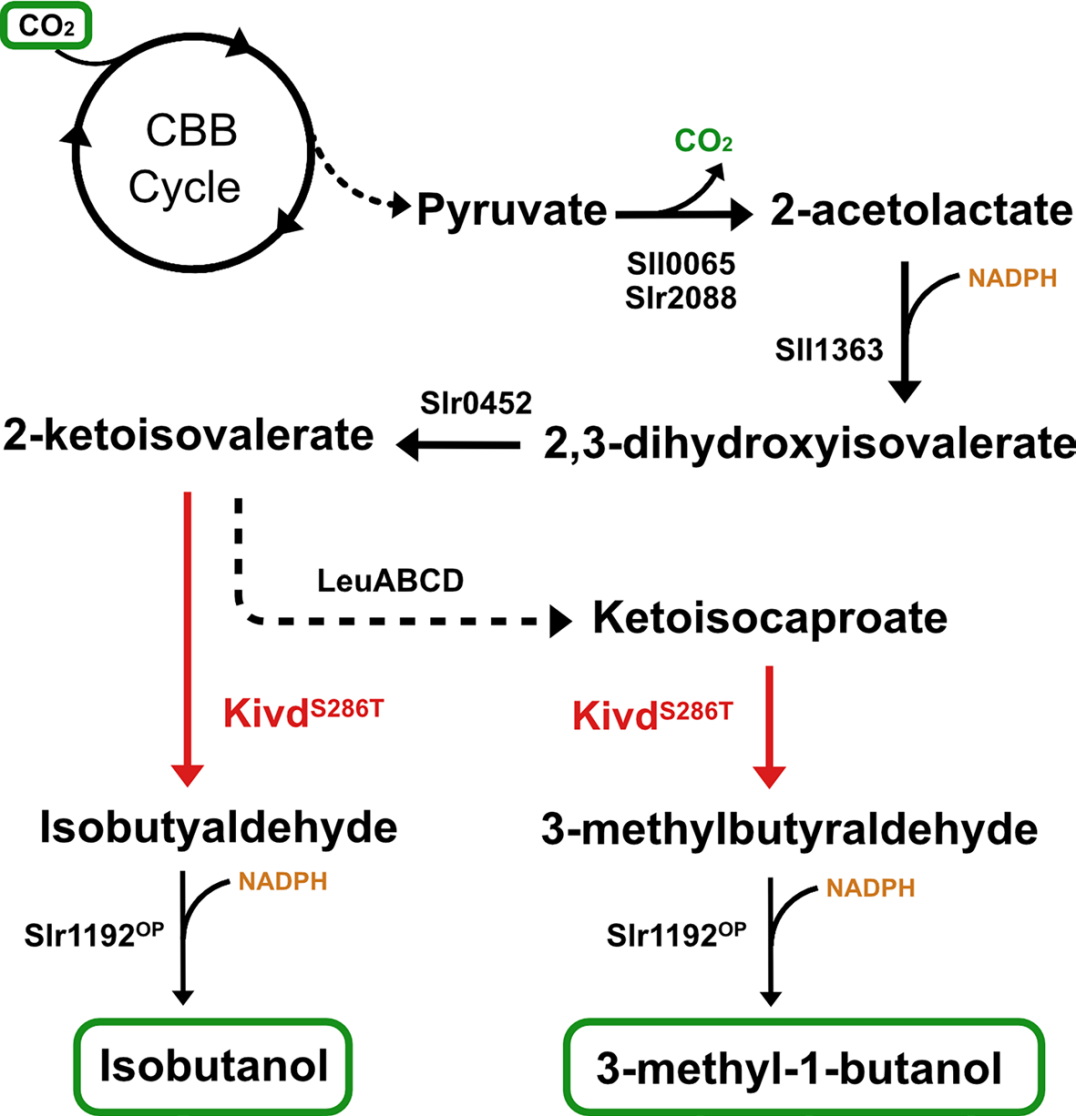
Isobutanol (IB) and 3-methyl-1-butanol (3M1B) biosynthesis pathway. Carbon dioxide is fixed by the Calvin–Benson–Bassham (CBB) cycle, and the fixed carbon flows into the 2-keto acid pathway for IB and 3M1B biosynthesis. Endogenous enzymes are written in black, while heterologous enzymes are written in red. Abbreviations of enzymes: Sll0065, small subunit of native acetolactate synthase (AlsS); Slr2088, large subunit of native AlsS; Sll1363, native acetohydroxy-acid isomeroreductase (IlvC); Slr0452, native dihydroxy-acid dehydratase (IlvD); LeuA, 2-isopropylmalate synthase; LeuCD, 3-isopropylmalate dehydratase; LeuB, 3-isopropylmalate dehydrogenase; Kivd^S286T^, α-ketoisovalerate decarboxylase (*Lactococcus lactis*); Slr1192^OP^, codon-optimized native alcohol dehydrogenase. Dotted lines indicate multiple reactions

Biological IB production was first demonstrated in *Escherichia coli* (*E. coli*) by introduction of a synthetic 2-keto acid pathway [7]. The 2-keto acid pathway involves five enzymes for IB biosynthesis from the central metabolite pyruvate (Fig. 1). Within the 2-keto acid pathway, the first involved enzyme, acetolactate synthase (AlsS), condenses two pyruvate molecules into a 2-acetolactate molecule. The 2-acetolactate is further converted to 2-ketoisovalerate by sequential enzymatic reactions catalyzed by acetohydroxy-acid isomeroreductase (IlvC) and dihydroxy-acid dehydratase (IlvD). As an intermediate for valine and leucine biosynthesis, 2-ketoisovalerate is decarboxylated by a heterologously expressed broad-substrate-range α-ketoisovalerate decarboxylase (Kivd) to isobutyaldehyde, and subsequently reduced into IB by an alcohol dehydrogenase (Adh). On the basis of the first report, the same strategy was applied in various microorganisms for IB biosynthesis [8, 9]. Meanwhile, due to the existence of native leucine biosynthesis pathway, 2-ketoisovalerate is converted into ketoisocaproate by sequential enzymes, encoded by *leuABCD*. The resulting ketoisocaproate is decarboxylated and reduced into 3-methyl-1-butanol (3M1B), by Kivd and Adh (Fig. 1). Similar to IB, 3M1B is a superior candidate for gasoline additive and is widely used as a precursor for various chemical synthesis [10].

Different from heterotrophic microorganisms feeding on substrates generated from plant biomass, photosynthetic microorganisms, including cyanobacteria, are capable to use sunlight and carbon dioxide for biofuel synthesis in a direct process. In that regard, the 2-keto acid pathway was successfully introduced into cyanobacteria for IB biosynthesis, first reported in *Synechococcus elongatus* PCC 7942 [11]. Thereafter, another model cyanobacterial strain, *Synechocystis* sp. PCC 6803 (*Synechocystis*) was demonstrated to have the ability to produce IB after a single heterologous expression of Kivd, originating from *Lactococcus lactis* [12, 13]. Furthermore, a by-product 3M1B was produced simultaneously with Kivd expression [12]. Protein engineering was performed on the key enzyme Kivd and a single replacement of Serine286 with Threonine significantly improved the Kivd activity further contributing towards an improved IB and 3M1B production [14]. This engineered Kivd^S286T^ has been used throughout following studies. In a more recent study, photosynthetic IB production was further enhanced by either increased Kivd^S286T^ expression level or integration of a complete 2-keto acid pathway [15]. Even with substantial progress reported on 2-keto acid pathway for photosynthetic IB and 3M1B production, the achieved production is still far behind to that of heterotrophic microorganisms [10] or cell-free system using a synthetic biochemistry approach [16]. Due to the low IB concentration in the cultivation broth and its azeotropic nature, downstream IB separation and purification require specific equipment with high energy consumption. Rectification is currently a widely used method for IB separation and purification [17]. Additional methods are available for separation of IB from the cultivation broth, such as gas stripping, pervaporation, vacuum evaporation, absorption, solvent extraction, salting-out and salting-out extraction [17].

In the present work, selected approaches were employed on the cyanobacterial strain *Synechocystis* to extensively explore the 2-keto acid pathway for IB and 3M1B biosynthesis. Firstly, two selected strains, HX29 and HX42, were cultivated continuously for 60 days in long-term milking experiments to explore their full capacities of IB and 3M1B biosynthesis. Secondly, additional efforts were invested to address if Kivd^S286T^ is still the bottleneck restraining further improvement of IB and 3M1B production by modifying the *kivd*^*S286T*^ copy number. Thirdly, instead of overexpressing heterologous enzymes, selected native enzymes involved in the valine/leucine biosynthesis were overexpressed to explore their effects on IB and 3M1B biosynthesis. Lastly, selective overexpression of genes involving in central carbon metabolism was experimentally verified to have positive contributions towards IB and 3M1B biosynthesis through the 2-keto acid pathway in *Synechocystis*. The collectively acquired information in this study further guides metabolic engineering strategies towards photosynthetic pyruvate-derived bioproduction.

## Materials and methods

### Genetic constructs

All plasmids used for generating engineered *Synechocystis* sp. PCC 6803 (*Synechocystis*) strains are listed in Additional file 1: Table S1. *Escherichia coli* (*E. coli*) strains DH5α-Z1 (Invitrogen) and T7 Express (NEB) were used for propagation of all plasmids used in this study. The *E. coli* strains were routinely cultivated in liquid lysogeny broth (LB) medium or 1.25% LB agar plates at 37 °C, with proper antibiotics supplemented. The final concentrations for different antibiotics were: spectinomycin, 50 μg mL^−1^ (AppliChem); kanamycin, 50 μg mL^−1^ (Thermo Fisher Scientific); chloramphenicol, 35 μg mL^−1^ (Sigma-Aldrich); and erythromycin, 200 μg mL^−1^ (Sigma-Aldrich). The self-replicating plasmid used was constructed previously [14]. All integrative plasmids were constructed based on the pEERM plasmid [18]. The homologous recombination regions are the around 1000 bp upstream sequence and 1000 bp downstream sequence of the integrative site of *Synechocystis* chromosome and were amplified from *Synechocystis* genomic DNA using specific primers (see Additional file 1: Table S2). The gene fragments of *pckA* and *tpiA* were amplified from *E. coli* DH5α genomic DNA with specific primers (see Additional file 1: Table S2). The sequences of gene fragments *kivd*^*S286T*^, *slr1192*^*OP*^, *sll0065*, *slr2088*, *sll1363*, *slr0452*, *alsS*, *ilvC*, *ilvD*, *fbaA*, *tktA* and *pyk1* were codon-optimized and synthesized by GenScript. All gene sequences and all primers used for plasmid construction are listed in Additional file 1: Table S2 and Table S3.

### Transformation methods for *Synechocystis* sp. PCC 6803

Natural transformation of integrative plasmids and conjugation of self-replicating plasmids were performed as described previously [15]. All generated engineered *Synechocystis* strains in this study are listed in Table 1.

**Table 1:** List of *Synechocystis* sp. PCC 6803 strains used in this study

Strain	Relevant genotypes^a^	References
WT	*Wild-type Synechocystis sp. PCC 6803*	[12]
HX29	*Δddh*::(P*trc*BCD-***kivd***^***S286T***^-T)-Cm^R^, *Δsll1564*::(P*trc*BCD-***kivd***^***S286T***^-T)-Sp^R^, pEEK2-(P*trc*BCD-***kivd***^***S286T***^-T)-Km^R^	[15]
HX42	*Δddh*::(P*trc*BCD-***slr1192***^***OP***^-***alsS***-T)-Cm^R^, *Δslr0168*::(P*trc*BCD-***ilvC***-***ilvD***-T)-Sp^R^, pEEK2-(P*trc*BCD-***kivd***^***S286T***^-T)-Km^R^	[15]
HX56	*Δddh*::(P*trc*BCD-***kivd***^***S286T***^-T)-Cm^R^, *Δsll1564*::(P*trc*BCD-***kivd***^***S286T***^-T)-Sp^R^, *Δslr0168*::(P*trc*BCD-***kivd***^***S286T***^-T)-Em^R^, pEEK2-(P*trc*BCD-***kivd***^***S286T***^-T)-Km^R^	This study
HX61	*Δddh*::(P*trc*BCD-***kivd***^***S286T***^-T)-Cm^R^, *Δsll1564*::(P*trc*BCD-***kivd***^***S286T***^-T)-Sp^R^, *Δslr0168*::Em^R^, pEEK2-(P*trc*BCD-***kivd***^***S286T***^-T)-Km^R^	This study
HX62	*Δddh*::(P*trc*BCD-***kivd***^***S286T***^-T)-Cm^R^, *ΔPEPc*::(P*trc*BCD-***kivd***^***S286T***^-T)-Sp^R^, *Δslr0168*::(P*trc*BCD-***kivd***^***S286T***^-T)-Em^R^, pEEK2-(P*trc*BCD-***kivd***^***S286T***^-T)-Km^R^	This study
HX63	*Δddh*::(P*trc*BCD-***kivd***^***S286T***^-T)-Cm^R^, *Δslr0186*::(P*trc*BCD-***kivd***^***S286T***^-T)-Sp^R^, *Δslr0168*::(P*trc*BCD-***kivd***^***S286T***^-T)-Em^R^, pEEK2-(P*trc*BCD-***kivd***^***S286T***^-T)-Km^R^	This study
HX74	*Δddh*::(P*trc*BCD-***slr1192***^***OP***^-***alsS***-T)-Cm^R^, *Δslr0168*::(P*trc*BCD-***ilvC***-***ilvD***-T)-Sp^R^, *ΔNSII*::(P*trc*BCD-***fbaA***-***tktA***-T)-Em^R^, pEEK2-(P*trc*BCD-***kivd***^***S286T***^-T)-Km^R^	This study
HX75	*Δddh*::(P*trc*BCD-***slr1192***^***OP***^-***alsS***-T)-Cm^R^, *Δslr0168*::(P*trc*BCD-***ilvC***-***ilvD***-T)-Sp^R^, *ΔNSII*::Em^R^, pEEK2-(P*trc*BCD-***kivd***^***S286T***^-T)-Km^R^	This study
HX77	*Δddh*::(P*trc*BCD-***slr1192***^***OP***^-***alsS***-T)-Cm^R^, *Δslr0168*::(P*trc*BCD-***ilvC***-***ilvD***-T)-Sp^R^, *ΔNSII*::(P*trc*BCD-***pckA***-***tpiA***-T)-Em^R^, pEEK2-(P*trc*BCD-***kivd***^***S286T***^-T)-Km^R^	This study
HX78	*Δddh*::(P*trc*BCD-***kivd***^***S286T***^-T)-Cm^R^, *Δslr1934*::(P*trc*BCD-***kivd***^***S286T***^-T)-Sp^R^, *Δslr0168*::(P*trc*BCD-***kivd***^***S286T***^-T)-Em^R^, pEEK2-(P*trc*BCD-***kivd***^***S286T***^-T)-Km^R^	This study
HX79	*Δddh*::(P*trc*BCD-***kivd***^***S286T***^-T)-Cm^R^, *Δslr0168*::(P*trc*BCD-***kivd***^***S286T***^-T)-Sp^R^, *ΔNSII*::Em^R^, pEEK2-(P*trc*BCD-***kivd***^***S286T***^-T)-Km^R^	This study
HX80	*Δddh*::(P*trc*BCD-***kivd***^***S286T***^-T)-Cm^R^, *Δsll1721*::(P*trc*BCD-***kivd***^***S286T***^-T)-Sp^R^, *Δslr0168*::(P*trc*BCD-***kivd***^***S286T***^-T)-Em^R^, pEEK2-(P*trc*BCD-***kivd***^***S286T***^-T)-Km^R^	This study
HX81	*Δddh*::(P*trc*BCD-***kivd***^***S286T***^-T)-Cm^R^, *Δslr0168*::(P*trc*BCD-***kivd***^***S286T***^-T)-Sp^R^, *ΔNSII*::Em^R^, pEEK2-(P*trc*BCD-***kivd***^***S286T***^-T)-Km^R^	This study
HX86	*Δddh*::(P*trc*BCD-***kivd***^***S286T***^-T)-Cm^R^, *Δslr0168*::(P*trc*BCD-***kivd***^***S286T***^-T)-Sp^R^, *ΔNSII*::(PtrcBCD-***pyk1***-***pckA***-T)-Em^R^, pEEK2-(P*trc*BCD-***kivd***^***S286T***^-T)-Km^R^	This study
HX87	*Δddh*::(P*trc*BCD-***kivd***^***S286T***^-T)-Cm^R^, *Δslr0168*::(P*trc*BCD-***kivd***^***S286T***^-T)-Sp^R^, *ΔNSII*::(P*trc*BCD-***pckA***-***tpiA***-T)-Em^R^, pEEK2-(P*trc*BCD-***kivd***^***S286T***^-T)-Km^R^	This study
HX88	*Δddh*::(P*trc*BCD-***slr1192***^***OP***^-***sll0065***-T)-Cm^R^, *Δslr0168*::(P*psbA2*-***sll1363***-***slr0452***-T)-Sp^R^, pEEK2-(P*trc*BCD-***kivd***^***S286T***^-T)-Km^R^	This study
HX89	*Δddh*::(P*trc*BCD-***slr1192***^***OP***^-***sll0065***-T)-Cm^R^, *Δslr0168*::(P*trc*BCD-***sll1363***-***slr0452***-T)-Sp^R^, pEEK2-(P*trc*BCD-***kivd***^***S286T***^-T)-Km^R^	This study
HX91	*Δddh*::(P*trc*BCD-***slr1192***^***OP***^-***slr2088***-***sll0065***-T)-Cm^R^, *Δslr0168*::(P*trc*BCD-***sll1363***-***slr0452***-T)-Sp^R^, pEEK2-(P*trc*BCD-***kivd***^***S286T***^-T)-Km^R^	This study

Expressed genes in bold

^a^Km^R^, kanamycin resistance cassette; Sp^R^, spectinomycin resistance cassette; Cm^R^, chloramphenicol resistance cassette; Em^R^, erythromycin resistance cassette; T, terminator BBa_B0015

### *Synechocystis* sp. PCC 6803 cultivation

*Synechocystis* seed cultures were routinely cultivated and maintained in liquid BG11 medium [19] or 1.25% BG11 agar plates under 30 μmol photons m^−2^ s^−1^ at 30 °C, with proper antibiotics supplemented. The final concentrations of antibiotics used were: spectinomycin, 25 μg mL^−1^; kanamycin, 25 μg mL^−1^; chloramphenicol, 10 μg mL^−1^; and erythromycin, 25 μg mL^−1^.

#### Cultivation condition of long-term milking experiments

Seed cultures were grown under 30 μmol photons m^−2^ s^−1^ at 30 °C in BG11 with appropriate antibiotic(s) in 100 mL Erlenmeyer flasks (VWR) until OD_750_ = 1.5–2.0. The seed cultures were then used to inoculate 25 mL experimental cultures to OD_750_ = 0.1 in BioLite 25 cm^2^ plug-sealed tissue culture flasks (Thermo Fisher Scientific). The medium used for experimental cultures was BG11 with addition of 50 mM NaHCO_3_ (Sigma-Aldrich) and appropriate antibiotic(s) (final concentrations: chloramphenicol, 10 μg mL^−1^; spectinomycin, 25 μg mL^−1^; erythromycin, 25 μg mL^−1^; and kanamycin, 25 μg mL^−1^). All experimental cultures were prepared in quadruplicates. The flasks were shaken horizontally at 120 rpm, under 50 μmol photons m^−2^ s^−1^ at 30 °C. Two milliliters of culture were sampled from each flask every second day for measurements and 2 mL of fresh BG11 medium with addition of 500 mM NaHCO_3_ (Sigma-Aldrich) and appropriate antibiotic(s) were added back. The pH of experimental cultures was measured with MColorpHast™ pH-indicator strips (pH 6.5–10) (Merck) and the cultures pH were adjusted to the range between 7–8 using 37% HCl (Sigma-Aldrich).

#### Cultivation condition of short-term screening experiments

Short-term screening experiments were performed as described before [15].

### Crude protein extraction and SDS-PAGE/Western-immunoblot

Crude protein extraction and SDS-PAGE/Western-immunoblot were performed as previously detailed [15]. Ten micrograms (Strep-tagged proteins) and 20 μg (His-tagged and Flag-tagged proteins) of soluble crude proteins were loaded for protein expression analysis.

### Optical density measurement

The cell growth of each culture was monitored by measuring optical density at 750 nm (OD_750_), as previously detailed [15].

### Products analysis

Isobutanol (IB) and 3-methyl-1-butanol (3M1B) were extracted from *Synechocystis* cultures every second day as described previously [15]. IB and 3M1B were analyzed using a PerkinElmer GC 580 system equipped with a flame ionization detector and an Elite-WAX Polyethylene Glycol Series Capillary column, 30 m × 0.25 mm × 0.25 μm (PerkinElmer). The detailed analytical program can be found in [12, 15]. In-flask titer is the IB and 3M1B titer directly measured from the culture; cumulative titer takes into account the dilution factor due to the harvesting/nutrient feeding every second day, i.e., the total production from the cell culture.

## Results and discussion

### Long-term milking experiments of *Synechocystis* sp. PCC 6803 strains HX29 and HX42

**Fig. 2:**
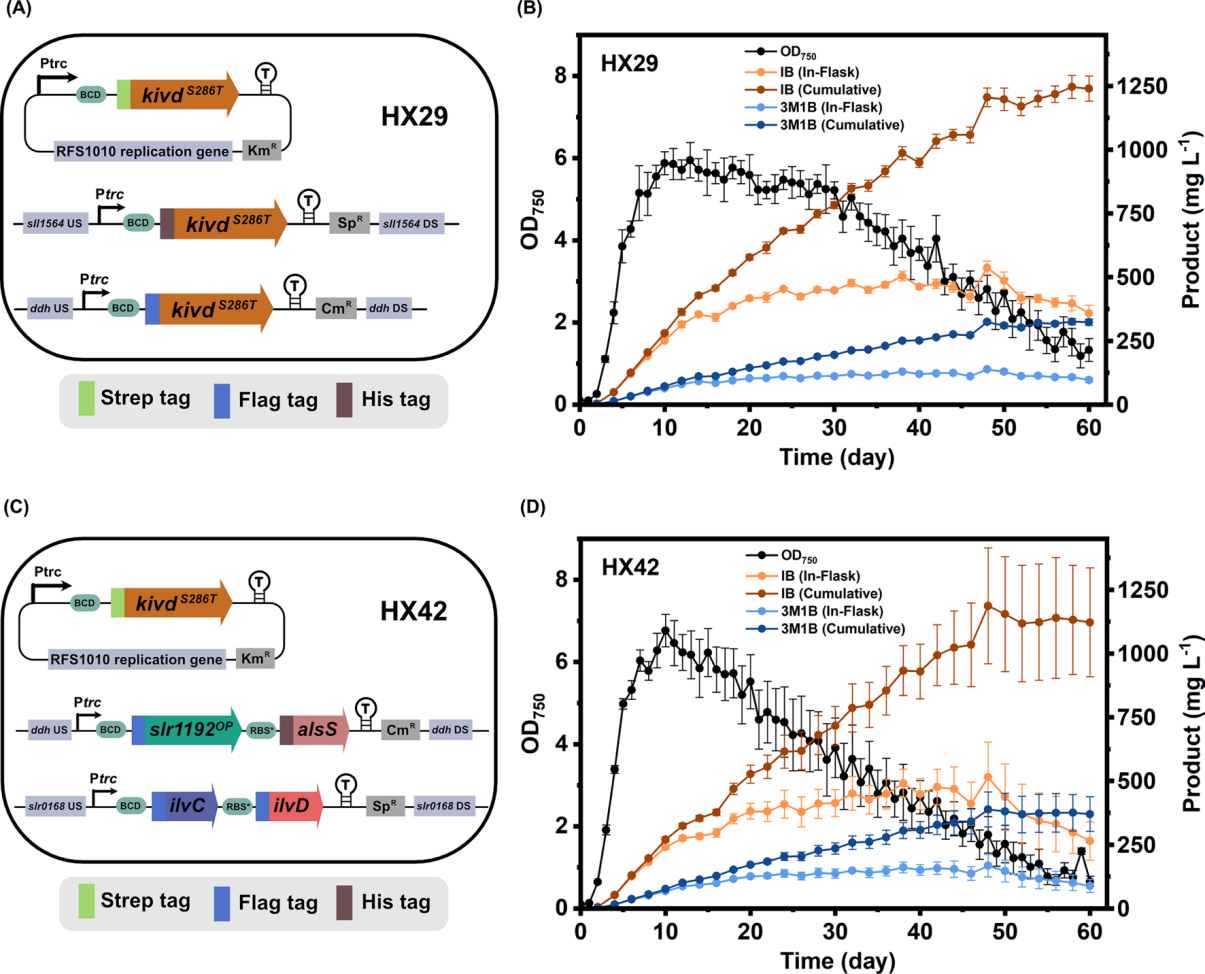
Long-term milking experiments of engineered *Synechocystis* sp. PCC 6803 strains HX29 and HX42. **A** Schematic diagram of plasmids used for generating strain HX29. *kivd*^*S286T*^: encodes α-ketoisovalerate decarboxylase (*Lactococcus lactis*). **B** Growth profile, isobutanol (IB) and 3-methyl-1-butanol (3M1B) in-flask and cumulative titers of strain HX29. **C** Schematic diagram of plasmids used for generating strain HX42. *kivd*^*S286T*^, encodes α-ketoisovalerate decarboxylase (*L. lactis*); *alsS*, encodes acetolactate synthase (*Bacillus subtilis*); *ilvC*, encodes acetohydroxy-acid isomeroreductase (*Escherichia coli*); *ilvD*, encodes dihydroxy-acid dehydratase (*E. coli*); *slr1192*^*OP*^, encodes codon-optimized alcohol dehydrogenase (*Synechocystis*). **D** Growth profile, IB and 3M1B in-flask and cumulative titers of strain HX42. Results are the mean of four biological replicates, each with three technical replicates. Error bars represent standard deviation

HX29 [15] is an engineered *Synechocystis* sp. PCC 6803 (*Synechocystis*) strain containing three copies of *kivd*^*S286T*^: one copy integrated into the *ddh* site of *Synechocystis* chromosome; the second copy integrated into the *sll1564* site; and the third copy placed on a self-replicating plasmid (Fig. 2A). In our previous study, strain HX29 showed the highest isobutanol (IB) production per cell among numerous IB-producing *Synechocystis* strains [15]. Therefore, long-term cultivation was performed on strain HX29 to characterize its full capacity of IB and 3-methyl-1-butanol (3M1B) production. The whole cultivation period lasted for sixty days, and the maximum optical density (OD_750_) of the experimental culture reached 5.95 on day 13 (Fig. 2B). The highest in-flask IB and 3M1B titers obtained of strain HX29 were 536.9 mg L^−1^ and 138.7 mg L^−1^, respectively, on day 48 (Fig. 2B). After day 48, the measured in-flask titers for IB and 3M1B started to decrease (Fig. 2B). In the end of the 60-day cultivation, the cumulative titers of using HX29 were 1247 mg L^−1^ and 326 mg L^−1^ for IB and 3M1B, respectively (Fig. 2B). By dividing the whole cultivation into six stages, the IB and 3M1B production rate is summarized in Table 2. In consistence with previous report [20], the highest in-flask production rate was observed in Stage I, corresponding to exponential phase, which is significantly higher than the other stages (Table 2). The highest cumulative production rate observed was in Stage II for both IB and 3M1B (Table 2).

**Table 2:** Isobutanol (IB) and 3-methyl-1-butanol (3M1B) production rates of engineered *Synechocystis* sp. PCC 6803 strains HX29 and HX42

	**HX29**				**HX42**			
**Growth stage**	**IB** (mg L^−1^ day^−1^)		**3M1B** (mg L^−1^ day^−1^)		**IB** (mg L^−1^ day^−1^)		**3M1B** (mg L^−1^ day^−1^)	
	In-flask	Cumulative	In-flask	Cumulative	In-flask	Cumulative	In-flask	Cumulative
Stage I (day 0–10)	**25.1 ± 0.6**	28.0 ± 0.6	**6.4 ± 0.1**	7.2 ± 0.1	**24.1 ± 1.0**	**27.1 ± 1.0**	**6.8 ± 0.1**	7.7 ± 0.1
Stage II (day 10–20)	16.6 ± 0.8	**29.8 ± 1.0**	3.9 ± 0.2	**7.3 ± 0.2**	14.0 ± 2.2	25.6 ± 2.6	5.8 ± 0.6	**9.5 ± 0.5**
Stage III (day 20–30)	3.1 ± 0.3	20.5 ± 0.4	0.8 ± 0.1	5.1 ± 0.1	3.2 ± 2.4	18.9 ± 4.2	1.1 ± 1.3	6.4 ± 1.8
Stage IV (day 30–40)	1.5 ± 0.7	20.3 ± 0.8	0.9 ± 0.2	5.6 ± 0.2	3.5 ± 1.3	21.3 ± 3.3	1.4 ± 0.4	7.3 ± 1.1
Stage V (day 40–50)	2.4 ± 2.4	21.2 ± 2.9	0.9 ± 0.6	5.9 ± 0.8	1.4 ± 6.3	20.8 ± 10.1	0.4 ± 2.0	6.8 ± 3.2
Stage VI (day 50–60)	− 12.8 ± 0.6	4.2 ± 0.9	3.2 ± 0.1	1.3 ± 0.3	− 18.7 ± 5.9	− 3.3 ± 1.7	− 6.3 ± 1.9	− 1.1 ± 0.5

The highest IB and 3M1B in-flask and cumulative production rate of each strain is shown in bold. Results are the mean of four biological replicates, each with three technical replicates. Errors represent standard deviation

In parallel, HX42 [15], a strain with a complete 2-keto acid pathway integrated, was cultivated under the same condition as strain HX29. The engineered strain HX42 contains the following genetic modifications: *slr1192*^*OP*^ and *alsS* integrated into the *ddh* site of *Synechocystis* chromosome; *ilvC* and *ilvD* integrated into the *slr0168* site; and *kivd*^*S286T*^ placed on a self-replicating plasmid (Fig. 2C). The growth curve showed a maximum OD_750_ of 6.77 on day 10 (Fig. 2D). The highest in-flask titers of IB and 3M1B of strain HX42 observed were 515.4 mg L^−1^ and 168.2 mg L^−1^, respectively (Fig. 2D). The final resulting cumulative IB and 3M1B titers of strain HX42 were 1155 mg L^−1^ and 389 mg L^−1^, respectively (Fig. 2D).

Both strains HX29 and HX42 achieved significantly higher cumulative IB and 3M1B titers compared to the previously best-performing strain [20] under the same cultivation conditions (Fig. 2B, D). The new records of IB and 3M1B cumulative titers were improved 1.4-fold and 1.7-fold by HX29 and HX42, respectively. A comprehensive comparison was performed between strains HX29 and HX42 from growth pattern to IB and 3M1B production. Strain HX42 grew faster in Stage I, while the OD_750_ declined faster after day 10 (Fig. 2B, D). Interestingly, strain HX42 showed lower in-flask/cumulative IB titer but higher in-flask/cumulative 3M1B titer (Fig. 2B, D). Different from HX29, the highest IB cumulative production rate of HX42 was observed in Stage I (Table 2), which may result from the different growth pattern between the two strains. As noted, a relatively large error bar of production curve indicates a relatively large variation among biological replicates of strain HX42. Among the four biological replicates, one of them grew much better than the rest and kept higher optical density (OD_750_) for a longer time-period, resulting in final cumulative titers of IB and 3M1B up to 1449 mg L^−1^ and 469 mg L^−1^. One possible variation source leading to the varied growth and IB and 3M1B production may be the unavoidable variation in the HCl titration procedures, as culture pH was controlled manually by acid titration and monitored through color indication of MColorpHast™ pH-indicator strips, making it difficult to maintain a precisely controlled culture pH among biological replicates.

For further improvement, a controlled cultivation system is an approach to achieve even higher IB and 3M1B titers. With a photobioreactor system equipped a pH controller, it will be possible to achieve maximum carbon assimilation efficiency and optimal growth rate. Moreover, considering that the highest production rate for IB and 3M1B was observed between day 0–20 during the long-term cultivation (Table 2), the second approach to further enhance products titer is to employ a re-inoculation strategy [21] with a cycle of e.g., 20 days, aiming to maintain in highest production rate throughout the cultivation period. In addition, final optimization could be achieved by testing various cultivation parameters, such as light intensity and quality, CO_2_ feeding and amount.

### Short-term screening experiments of newly constructed engineered *Synechocystis* sp. PCC 6803 strains

#### Generating engineered *Synechocystis* sp. PCC 6803 strains containing a complete native 2-keto acid pathway

**Fig. 3:**
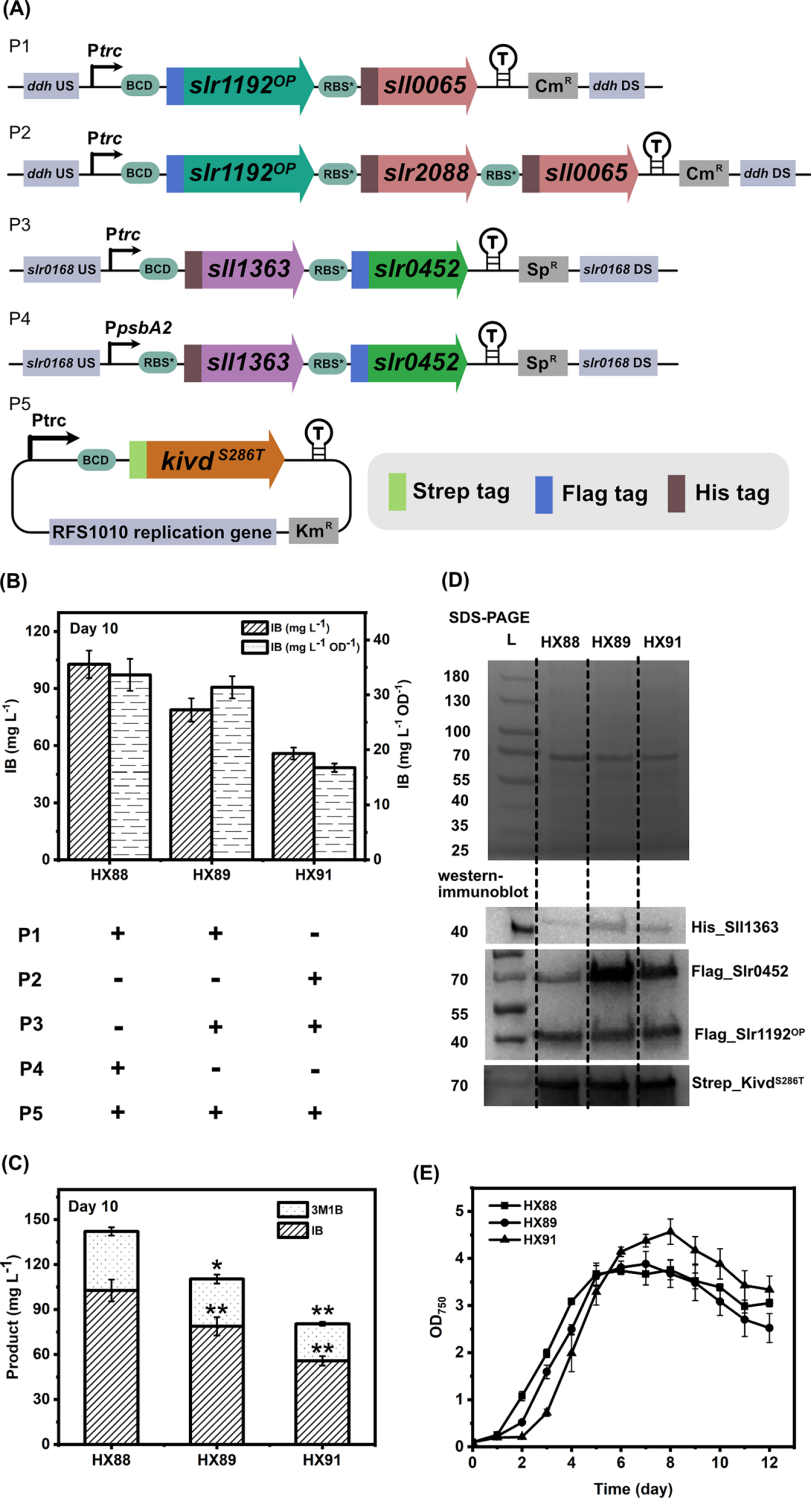
Generation and analysis of engineered *Synechocystis* sp. PCC 6803 strains with a complete native 2-keto acid pathway integration. **A** Schematic diagram of plasmids used to generate strains in Fig. 3B-E. P1 and P2 are integrative plasmids targeting *ddh* (*slr1556*) site of *Synechocystis* chromosome. P3 and P4 are integrative plasmids targeting *slr0168* site. P5 is a self-replicating plasmid. **B** The IB titer and IB production per cell on day 10 of engineered *Synechocystis* strains HX88, HX89 and HX91. Strain HX88 was generated by transformation with plasmids P1, P4 and P5; strain HX89 was generated by transformation with plasmids P1, P3 and P5; strain HX91 was generated by transformation with plasmids P2, P3 and P5. **C** The IB and 3M1B titers on day 10 of engineered *Synechocystis* strains HX88, HX89 and HX91. **D** SDS-PAGE (top) and Western-immunoblot (bottom). L, ladder (in kDa). For SDS-PAGE, 10 μg of total soluble proteins were loaded for each strain. For Western-immunoblot, 10 μg, 20 μg, and 20 μg of total soluble proteins were loaded for each strain to detect Strep-tagged, Flag-tagged, and His-tagged proteins, respectively. Protein size: Kivd^S286T^, 61 kDa; Sll1363, 40 kDa; Slr0452, 59 kDa; Slr1192^OP^, 36 kDa. **E** Growth profile of engineered *Synechocystis* strains HX88, HX89 and HX91. Results are the mean of three biological replicates, each with three technical replicates. Error bars represent standard deviation. Asterisk represents significant difference between strains HX88 and HX89, or strains HX89 and HX91 (one-way ANOVA, * p < 0.05, ** p < 0.005)

In our previous report [15], we successfully constructed the engineered *Synechocystis* strain HX42 containing a complete 2-keto acid pathway consisting of four foreign enzymes and one native enzyme: acetolactate synthase (AlsS) from *Bacillus Subtilis*, acetohydroxy-acid isomeroreductase (IlvC) and dihydroxy-acid dehydratase (IlvD) from *Escherichia coli* (*E. coli*), α-ketoisovalerate decarboxylase (Kivd^S286T^) from *Lactococcus lactis*, and a codon-optimized alcohol dehydrogenase (Slr1192^OP^) from *Synechocystis*. In *Synechocystis*, it is still not settled which gene(s) encode(s) the native AlsS [20], though it was reported that three endogenous genes are potential candidates: *sll0065* encodes the regulatory subunit, while *slr2088* and *sll1981* encode the catalytic subunits [22]. The protein sequence of Sll1981 shares about 60% homology to AlsS from *B. subtilis* [23], while *slr2088*- and *sll0065*-encoded proteins are homologous to acetohydroxy-acid synthase (AHAS) [24]. Native IlvC and IlvD are encoded by *sll1363* and *slr0452*, respectively. Initially, different engineered strains with different combinations of native AlsS subunits were planned (data not shown), however, it was challenging to obtain correct transformants for most of them even after several attempts. In the end, only three engineered *Synechocystis* strains with a complete native 2-keto acid pathway (Fig. 1) were generated using four integrative plasmids and one self-replicating plasmid (Fig. 3A). Plasmid P1 was used to overexpress *slr1192*^*OP*^ and *sll0065* under the P*trc* promoter, while simultaneously knocking out the *ddh* gene. Likewise, plasmid P2 was used to overexpress *slr1192*^*OP*^, *slr2088*, and *sll0065*. Plasmids P3 and P4 were used to integrate *sll1363* and *slr0452* into the *slr0168* site under the control of P*trc* and P*psbA2* promoters, respectively. Plasmid P5 was a broad-host-range self-replicating plasmid for *kivd*^*S286T*^ expression.

Strain HX88, with a complete native 2-keto acid pathway integrated, produced 98 mg IB L^−1^ on day 10 (Fig. 3B, C), which is comparable to the IB titer obtained when using strain HX42 [15], indicating the native enzymes are as effective as the foreign enzymes for IB biosynthesis. All overexpressed proteins in HX88 were confirmed by SDS-PAGE/Western-immunoblot, except for Sll0065 (Fig. 3D).

It was observed that co-overexpressing IlvC and IlvD is a potential approach to channel more carbon flux into 2-keto acid pathway for IB production [20]. To further increase protein expression level, the promoter used to drive Sll1363 and Slr0452 expression was changed from P*psbA2* to the stronger P*trc* [25, 26], resulting in the engineered strain HX89 (Fig. 3A, B). As expected, the expression levels of Sll1363 and Slr0452 in HX89 increased compared to in the control strain HX88 (Fig. 3D). However, both IB and 3M1B titers were significantly lower in HX89 (Fig. 3C). As one possible explanation, the decreased titers may be due to the slower growth rate between days 0–5 (Fig. 3E), the time-period when majority of IB and 3M1B are produced. On the other hand, the observed IB and 3M1B titer difference demonstrates that the expression levels of Sll1363 and Slr0452 in strain HX88 are enough and not bottlenecks of the 2-keto acid pathway for IB and 3M1B biosynthesis.

Furthermore, as recently commented [27], the large catalytic subunit Slr2088 may form a complex with the small regulatory subunit Sll0065 to function as native AlsS in *Synechocystis*. However, this needs further validation. After several attempts, an engineered *Synechocystis* strain with co-overexpression of both the regulatory subunit and the catalytic subunit was generated (HX91). Compared to strain HX89, strain HX91 had a distinct growth pattern with a longer lag phase, barely any growth between days 0–2 (Fig. 3E). Thereafter, it caught up and reached a higher optical density (OD_750_ = 4.6) on day 8 (Fig. 3E). Unfortunately, the IB and 3M1B titers of HX91 were lower than that of HX89 (Fig. 3C), indicating that overexpressing Slr2088 has reverse effects on IB and 3M1B production. The mechanism of Slr2088 overexpression negatively affecting IB and 3M1B production is currently unknown. High-throughput-omics approaches [28, 29] may provide new insights to reveal this observation.

As shown in Fig. 3D, similar to HX88, the native AlsS was not detected by SDS-PAGE/ Western-immunoblot in strains HX89 and HX91. Several hypothesize could be made: *sll0065* and *slr2088* were successfully transcribed into mRNAs and were further translated to functional proteins, but their expression was too low to be detected by Western-immunoblot; or *sll0065* and *slr2088* were successfully transcribed into mRNAs, which were not translated into functional proteins due to some unknown native regulation; or the native *alsS* genes were expressed in specific growth phase and specific growth conditions, and unfortunately at the time of cell harvesting, there was no expression of native AlsS [23]. RT-PCR on the overexpressed genes encoding native AlsS will be the first step to validate the above hypotheses, and for further characterization, native AlsS could be defined by using a newly developed technique [30], followed by kinetic determinations.

#### ***kivd***^***S286T***^ copy number makes a significant difference for isobutanol and 3-methyl-1-butanol biosynthesis

**Fig. 4:**
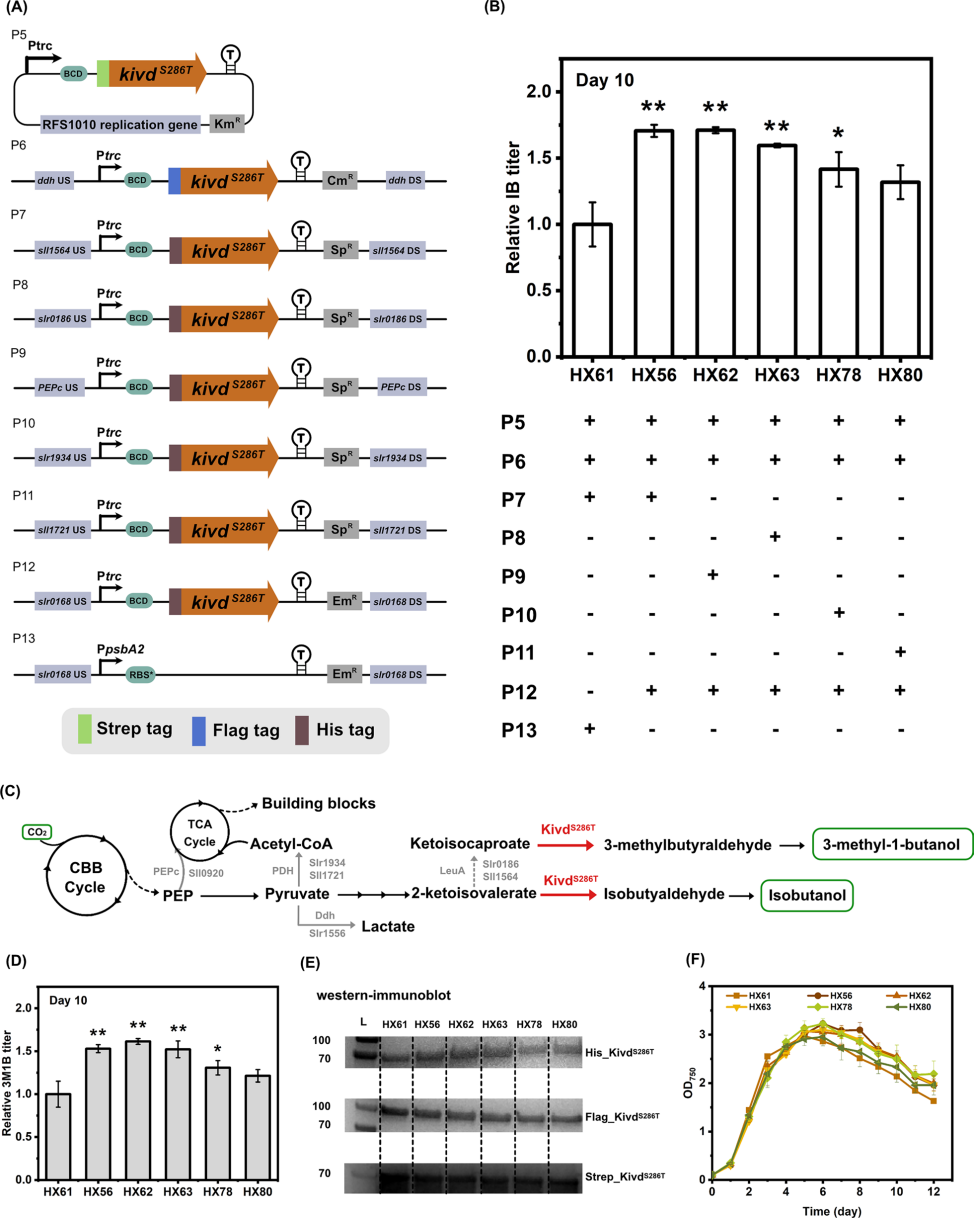
Engineered *Synechocystis* sp. PCC 6803 strains with four copies of *kivd*^*S286T*^ significantly improved isobutanol (IB) and 3-methyl-1-butanol (3M1B) titers compared to control strain with three copies of *kivd*^*S286T*^. **A** Schematic diagram of plasmids used to generate strains in Fig. 4. P5 is a self-replicating plasmid; P6-P13 are integrative plasmids targeting various sites of *Synechocystis* chromosome. **B** The relative IB titer of engineered *Synechocystis* strains HX56, HX61-63, HX78 and HX80. **C** Simplified pathway for IB and 3M1B biosynthesis. Endogenous enzymatic reactions are written in black; heterologous enzymatic reactions are written in red; knock-out/knock-down enzymatic reactions are written in grey. Abbreviations of enzymes: Kivd^S286T^, α-ketoisovalerate decarboxylase (*Lactococcus lactis*); PEPc (encoded by *sll0920*), phosphoenolpyruvate carboxylase; PDH (encoded by *slr1934* and *sll1721*), pyruvate dehydrogenase E1 component; Ddh (encoded by *slr1556*), D-lactate dehydrogenase; LeuA (encoded by *slr0186* and *sll1564*), 2-isopropylmalate synthase. Abbreviations of intermediates: PEP, phosphoenolpyruvate. **D** The relative 3M1B titer of engineered *Synechocystis* strains HX56, HX61-63, HX78 and HX80. **E** Western-immunoblot analysis of all expressed enzymes. L, ladder (in kDa). Ten micrograms, 20 μg, and 20 μg of total soluble proteins were loaded for each strain to detect Strep-tagged, Flag-tagged, and His-tagged proteins, respectively. Protein size: Kivd^S286T^, 61 kDa. **F** Growth profile of engineered *Synechocystis* strains HX56, HX61-63, HX78 and HX80. Results are the mean of three biological replicates, each with three technical replicates. Error bars represent standard deviation. Asterisk represents significant difference between engineered strains and control strain (one-way ANOVA, *p < 0.05, **p < 0.005)

*kivd*^*S286T*^, encoding α-ketoisovalerate decarboxylase, is a verified critical enzyme for IB biosynthesis using *Synechocystis* as cell factory [14], and the IB titer improved in a stepwise manner with varied *kivd*^*S286T*^ copy number ranging from one copy to three copies [15]. In the current study, further attempt was pursued to increase *kivd*^*S286T*^ copy number. Initially, integrating plasmids P5, P6, P7 and P13 into wild-type *Synechocystis* resulted in a control strain HX61, containing three copies of *kivd*^*S286T*^ (Fig. 4A, B). P5 was constructed to express the first copy of *kivd*^*S286T*^ on self-replicating plasmid; P6 was constructed to integrate a second copy of *kivd*^*S286T*^ into the *ddh* site of *Synechocystis* chromosome; P7 was constructed to integrate a third copy of *kivd*^*S286T*^ into the *sll1564* site; P13 was constructed to integrate an erythromycin resistance cassette into the *slr0168* site (Fig. 4A). Then a base strain was generated by integrating plasmids P5, P6 and P12 (Fig. 4A), which was used to further construct *Synechocystis* strains with four copies of *kivd*^*S286T*^. Plasmid P12 was constructed to introduce one copy of *kivd*^*S286T*^ in the *slr0168* site (Fig. 4A). Engineered strains HX56, HX62, HX63, HX78 and HX80, containing four copies of *kivd*^*S286T*^, were generated by integrating plasmids P7, P9, P8, P10 and P11 into the base strain, respectively (Fig. 4A, B). Plasmids P7-11 were designed to integrate a fourth copy of *kivd*^*S286T*^ into the selected sites of *Synechocystis* chromosome (Fig. 4A, C). The rationale of the integration sites selection has been detailed previously [15].

Strain HX56, containing one more copy of *kivd*^*S286T*^ in the *slr0168* site, produced a 1.7-fold increased IB titer compared to the control strain HX61 (Fig. 4B). Similarly, the 3M1B titer was increased by 1.5-fold (Fig. 4D). The above results serve as evidence that the IB and 3M1B titers are positively correlated with *kivd*^*S286T*^ copy number, in consistence to the facts observed in a previous study [15]. Kivd^S286T^ expression of both strains was confirmed by Western-immunoblot (Fig. 4E) and growth profile was generated through measuring optical density (OD_750_) every day (Fig. 4F). Strain HX56 grew slower between days 0–4, while maintained a higher OD_750_ from day 5 and reached a higher maximum OD_750_ at 3.2 on day 6 (Fig. 4F).

To explore if the different integration sites of *Synechocystis* chromosome will make differences on growth and IB and 3M1B titers, four more strains were generated, named HX62, HX63, HX78 and HX80. As expected, all strains produced significantly higher IB and 3M1B titers than that produced by control strain HX61, except for strain HX80 (Fig. 4B, D). The fact that the IB and 3M1B titers of HX80 were not significantly improved may be caused by its deficient growth profile (Fig. 4F).

As a continuation study of our previous report [15], we successfully generated metabolically engineered *Synechocystis* strains containing four copies of *kivd*^*S286T*^. Taken together, Kivd^S286T^ is still the critical enzyme catalyzing the rate-limiting step of 2-keto acid pathway for IB and 3M1B biosynthesis. Currently, four antibiotics were used to screen for positive transformants containing four copies of *kivd*^*S286T*^, making it infeasible to further increase *kivd*^*S286T*^ copy number using traditional transformation approaches, since there is no report using more than four antibiotics for *Synechocystis* transformants screening and cultivation. As one of the solutions, marker-less genome editing strategy [31–33] may make it possible to generate engineered *Synechocystis* strains with higher *kivd*^*S286T*^ copy number. The marker-less-based CRISPR (clustered regularly interspaced short palindromic repeats) editing has been successfully applied in cyanobacteria for succinate production [34]. On the other hand, considering the time and efforts required for multiple transformation and selection procedures in marker-less genome editing approaches, instead of generating strains with multiple *kivd*^*S286T*^ copies, protein engineering will be a powerful alternative to improve the performance of Kivd^S286T^ enzyme on IB and 3M1B biosynthesis. Currently, Kivd^S286T^, an engineered version of wild-type Kivd after site-directed mutagenesis [14], was used throughout this study. Starting from Kivd^S286T^, directed evolution [35] may be employed to screen for superior Kivd variants, with further enhanced catalytic activity and/or specificity.

#### Identification of targets in central carbon metabolism for enhanced isobutanol and 3-methyl-1-butanol production

**Fig. 5:**
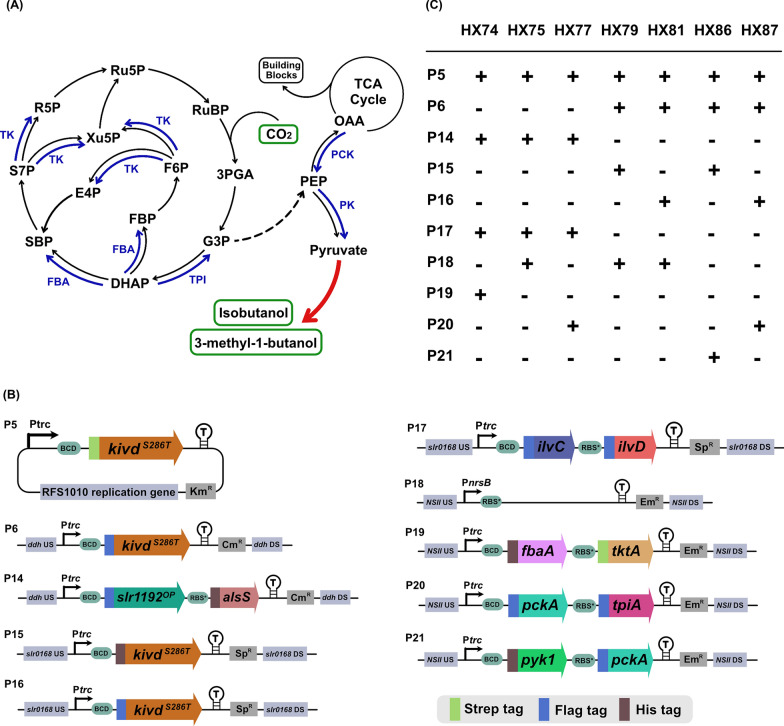
Schematic overview of metabolic engineering strategies adopted for isobutanol (IB) and 3-methyl-1-butanol (3M1B) production and the corresponding engineered *Synechocystis* sp. PCC 6803 strains. **A** Simplified pathway for IB and 3M1B biosynthesis. Endogenous pathways are written in black; heterologous pathways are written in red; targeting pathway for metabolic engineering are written in blue. Multiple enzymatic reactions are represented as dashed lines. Abbreviations of enzymes: FBA, aldolase (encoded by *fbaA*); TK, transketolase (encoded by *tktA*); PCK, phosphoenolpyruvate carboxykinase (encoded by *pckA*); TPI, triosephosphate isomerase (encoded by *tpiA*); PK, pyruvate kinase (encoded by *pyk1*). Abbreviations of intermediates: RuBP, ribulose-1,5-bisphosphate; 3PGA, 3-phosphoglycerate; G3P, glyceraldehyde-3-phosphate; DHAP, dihydroxyacetone phosphate; FBP, fructose-1,6-bisphosphate; SBP, sedoheptulose-1,7-bisphosphate; F6P, fructose-6-phosphate; S7P, sedoheptulose-7-phosphate; E4P, erythrose- 4-phosphate; Xu5P, xylulose-5-phosphate; R5P, ribose-5-phosphate; Ru5P, ribulose-5-phosphate; PEP, phosphoenolpyruvate; OAA, oxaloacetate. **B** Schematic diagram of plasmids used to generate *Synechocystis* strains in Fig. 5C and Fig. 6A–J. P5 is a self-replicating plasmid; P6, and P14-P21 are integrative plasmids targeting various sites of *Synechocystis* chromosome. **C** Genetic background of the engineered *Synechocystis* strains

Pyruvate is one of the central carbon compounds used as substrate for many cellular metabolite biosynthesis. IB and 3M1B are synthesized through pyruvate-derived 2-keto acid pathway. Apart from focusing on optimizing the 2-keto acid pathway itself, for the first time, various targets of the central carbon metabolism (Fig. 5A) were systematically evaluated for the effects on IB and 3M1B production in *Synechocystis*. The detailed information of the engineered strains is shown in Fig. 5 and Table 1. The genetic constructs designed to generate engineered *Synechocystis* strains are listed in Fig. 5B. Strains HX75, HX79 and HX81 serve as control strains. In detail, strain HX75 contains a complete 2-keto acid pathway consisting of four foreign enzymes and one native enzyme, while strains HX79 and HX81 contain three copies of *kivd*^*S286T*^.

**Fig. 6:**
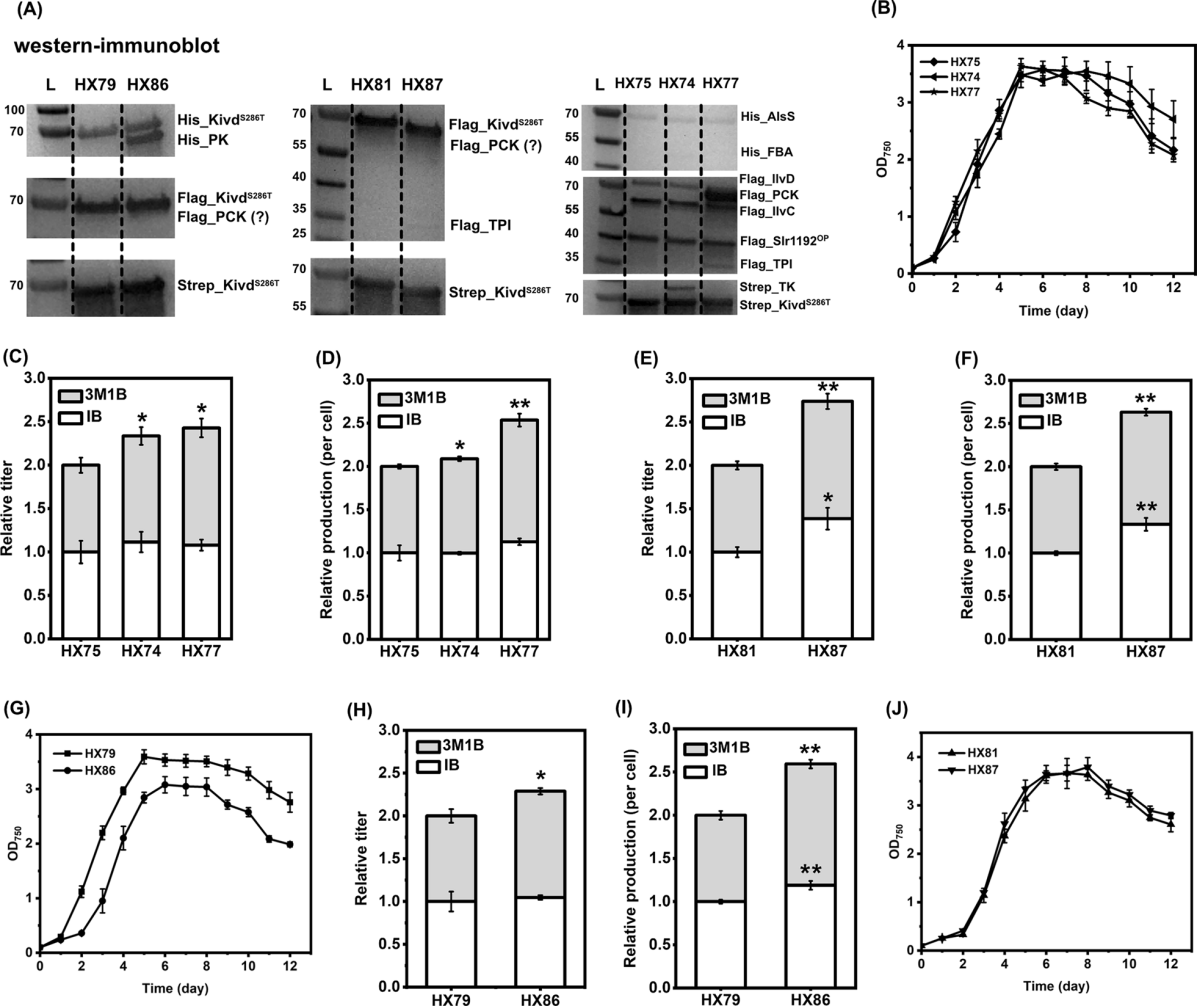
Positive effects of rewiring central carbon metabolism on photosynthetic isobutanol (IB) and 3-methyl-1-butanol (3M1B) production. **A** Western-immunoblot analysis of all overexpressed enzymes. L, ladder (in kDa). Ten micrograms, 20 μg, and 20 μg of total soluble proteins were loaded for each strain to detect Strep-tagged, Flag-tagged, and His-tagged proteins, respectively. Protein size: Kivd^S286T^, 61 kDa; PK, 53 kDa; PCK, 60 kDa; TPI, 27 kDa; AlsS, 62 kDa; FBA, 39 kDa; IlvD, 65 kDa; IlvC, 54 kDa; Slr1192^OP^, 36 kDa; TK, 72 kDa. **B** Growth profile of engineered *Synechocystis* sp. PCC 6803 strains HX74, HX75 and HX77. **C** Relative IB and 3M1B titers of the engineered *Synechocystis* strains HX74, HX75 and HX77 on day 10. **D** Relative IB and 3M1B production per cell of the engineered *Synechocystis* strains HX74, HX75 and HX77 on day 10. **E** Relative IB and 3M1B titers of the engineered *Synechocystis* strains HX81 and HX87 on day 10. **F** Relative IB and 3M1B production per cell of the engineered *Synechocystis* strains HX81and HX87 on day 10. **G** Growth profile of engineered *Synechocystis* strains HX79 and HX86. **H** Relative IB and 3M1B titers of the engineered *Synechocystis* strains HX79 and HX86 on day 10. **I** Relative IB and 3M1B production per cell of the engineered *Synechocystis* strains HX79 and HX86 on day 10. **J** Growth profile of engineered *Synechocystis* strains HX81 and HX87. Results are the mean of three biological replicates, each with three technical replicates. Error bars represent standard deviation. Asterisk represents significant difference between different strains (one-way ANOVA, * p < 0.05, ** p < 0.005)

The first two enzymes tested are aldolase (FBA) and transketolase (TK), which are involved in the Calvin–Benson–Bassham (CBB) cycle (Fig. 5A) and the oxidative pentose phosphate (OPP) or glycolysis pathway. Overexpression of FBA and TK has positive effects on cell growth as well as ethanol production in engineered *Synechocystis* strains [36, 37]. In *Synechocystis*, both class I and class II FBAs are present, encoded by *slr0943* and *sll0018*, respectively [24]. Class II FBA contributes to approximately 90% of total activity of the reversible alcohol condensation of dihydroxyacetone phosphate (DHAP) and glyceraldehyde 3-phosphate (G3P) [38]. Therefore, codon-optimized gene sequences of *sll0018* and *sll1070*, encoding class II FBA and TK, were synthesized, and used for building genetic constructs. An engineered strain HX74 was generated, with additional FBA and TK overexpression, when compared to the control strain HX75 (Fig. 5B, C). All overexpressed proteins were successfully identified through Western-immunoblot, though the band of FBA protein is barely visible (Fig. 6A). FBA expression was further verified by increasing the crude protein loading amount from 20 μg to 162 μg (Additional file 1: Fig. S1). A distinct growth difference between the two strains was observed after day 7, the OD_750_ of the control strain HX75 declined faster than strain HX74 (Fig. 6B). There was no significant difference of IB titer and IB production per cell between strains with or without FBA and TK co-overexpression (Fig. 6C, D). In contrast, a significant increase of 3M1B titer and 3M1B production per cell of HX74 was observed (Fig. 6C, D). The obtained improved 3M1B production may result from the critical roles of FBA and TK in ribulose-1,5-bisphosphate (RuBP) regeneration within the CBB cycle.

Apart from the CBB cycle as the main carbon assimilation machinery in *Synechocystis* with ribulose-1,5-bisphosphate carboxylase/oxygenase (RuBisCO) as the key carbon fixation enzyme, there is a second major carbon-fixing enzyme, named phosphoenolpyruvate carboxylase (PEPc). It was reported that 25% of inorganic carbon assimilation may be through the PEPc catalyzed reaction in *Synechocystis* under mixotrophic or heterotrophic conditions [39]. Phosphoenolpyruvate (PEP), one of the precursors for pyruvate synthesis, is converted by PEPc to generate oxaloacetate (OAA), which further feeds into the tricarboxylic acid cycle (TCA cycle) for building block biosynthesis. Complete knock-out of PEPc in *Synechocystis* is challenging due to its essential role in phototrophic growth in cyanobacteria [40]. Alternatively, heterologous expression of phosphoenolpyruvate carboxykinase (PCK) from *E. coli* is one of the approaches to partially eliminate the carbon flow from PEP to TCA cycle and channel more carbon flow towards pyruvate for IB and 3M1B biosynthesis (Fig. 5A). It was experimentally verified in *Synechococcus elongatus* PCC 7942 that PCK expression significantly improved the aldehyde production [41]. Moreover, under phototrophic conditions, the CBB cycle is the dominant pathway for carbon assimilation. G3P, an intermediate of CBB cycle, is a connection node between the CBB cycle and the glycolysis pathway. Within *Synechocystis* cells, assimilated carbon flows either from the CBB cycle or glycolysis pathway into G3P. Starting from G3P, pyruvate and acetyl-CoA are synthesized and involved in various complex metabolic pathways. It may be interesting to overexpress enzymes connecting two metabolites involved in both the CBB cycle and the glycolysis pathway, to cause perturbation of central carbon metabolism, which may have unexpected effects for pyruvate-derived product biosynthesis. It has been suggested that overexpression of triosephosphate isomerase* (*TPI) in *E. coli* could effectively enhance pyruvate-derived phloroglucinol production [42] by directing the glycolysis flux into pyruvate formation. Taken together, these two above-mentioned strategies may be promising to further enhance the IB and 3M1B biosynthesis in *Synechocystis*.

To test a combined effect of simultaneous expression of TPI and PCK, originating from *E. coli*, on IB and 3M1B production in *Synechocystis*, two engineered strains HX77 and HX87 were generated (Fig. 5B, C). HX77 was constructed by integrating *tpiA* and *pckA* in the neutral site II (NSII) [43] of strain HX42 [15], and strain HX87 was constructed by integrating *tpiA* and *pckA* in the NSII of strain HX28 [15]. In both strains, the expression of both genes was driven by the strong synthetic P*trc* promoter. Protein TPI expression was successfully identified by Western-immunoblot in both strains, whereas PCK expression was only confirmed in strain HX77 (Fig. 6A). The unsureness of PCK expression in strain HX87 is due to the similar estimated protein size of PCK and Kivd^S286T^, with less than 1.5 kDa difference. Based on the obtained results, two possible explanations may be made: PCK was successfully expressed, but the detected band overlapped with the band of Kivd^S286T^; or PCK was not expressed in the provided cultivation condition and harvesting time point. However, in strain HX87, PCK and TPI were expressed from a single operon (Fig. 5B) and the second gene in the operon, *tpiA*, was expressed successfully (Fig. 6A), suggesting that most probably the first gene in the operon was also successfully expressed. Further attempts were invested to explore an optimized condition for the Western-immunoblot, e.g., amount of crude protein loading, SDS-PAGE running conditions. Unfortunately, it is still challenging to visualize two clearly separated bands (Additional file 1: Fig. S1).

Strains HX75 and HX81 were generated as control strains for strains HX77 and HX87, respectively. After cultivated in short-term screening condition, strain HX77 produced significantly higher 3M1B titer and 3M1B production per cell by 1.3-fold and 1.4-fold, respectively, relative to the control strain HX75 (Fig. 6C, D). Meanwhile, the produced IB was only slightly improved in strain HX77, 1.1-fold, which is not statistically significant (Fig. 6C, D). On the other hand, when compared to the control strain HX81, strain HX87 accumulated significantly higher IB and 3M1B titers (Fig. 6E). Similarly, after normalized to optical density (OD_750_), IB and 3M1B production per cell of HX87 were both improved by 1.3-fold relative to strain HX81 (Fig. 6F). In conclusion, positive effects of a co-expression of PCK and TPI on photosynthetic IB and 3M1B production were experimentally verified using two different genetic backgrounds.

Metabolic engineering was successfully performed in the carbon fixation pathway as well as the pyruvate-derived 2-keto acid pathway. However, there is still space for further improvement through speeding up carbon flow between carbon fixation and the pyruvate-derived 2-keto acid pathway. In *Synechocystis*, pyruvate is synthesized from G3P in five enzymatic steps catalyzed by glyceraldehyde 3-phosphate dehydrogenase (Gap1), phosphoglycerate kinase (Pgk), 2,3-bisphosphoglycerate-independent phosphoglycerate mutase (Gpm), enolase (Eno), and pyruvate kinase (PK) (Additional file 1: Fig. S1). Singly overexpression of PK in *Synechococcus* resulted in significantly improved isobutyraldehyde production through the 2-keto acid pathway [44] and singly overexpression of Gpm or Eno also had positive effects on pyruvate-derived isoprene production in *Synechocystis* [45]. To further test if overexpression of Gpm, Eno and PK may promote IB and 3M1B production in *Synechocystis* and if there is any additive effect of overexpression of these enzymes, multiple plasmids were designed and generated (data not shown). However, there was an obstacle preventing further characterization, as it was impossible to acquire positive *Synechocystis* transformants after several attempts with traditional natural transformation methods. Developing novel genetic engineering tools and having them optimized and ready for generating engineered *Synechocystis* strains efficiently and precisely are in progress to overcome the encountered challenges.

Strain HX86, expressing PK and PCK, was constructed by integrating *pyk1* and *pckA* in the NSII of strain HX15 (Fig. 5B, C) [15]. Meanwhile, a control strain, HX79, was constructed by integrating an erythromycin resistance cassette in the NSII of strain HX15 (Fig. 5B, C). All expressed proteins were successfully identified and confirmed by Western-immunoblot (Fig. 6A), except for PCK, which was difficult to be separated from the band of Kivd^S286T^ due to similar expected protein size (Additional file 1: Fig. S1).

Strain HX86 grew significantly worse than control strain from three aspects: it had a longer lag phase in the beginning of cultivation; the OD_750_ of HX86 declined faster after day 8; and the measured OD_750_ was lower than that of control strain throughout the entire cultivation time-period (Fig. 6G). The observed severe growth retardation may be caused by PCK expression, similar to what was observed in an engineered *Synechococcus* strain with PCK expressed using a metal-inducible promoter [41]. Interestingly, the severe growth inhibition phenomenon was not observed for strains HX77 and HX87, both of which had PCK expressed. The cause of the different growth phenotype is currently unknown. Further detailed analysis, e.g., proteomics and metabolomics analysis, are needed to identify and clarify the cause. As shown in Fig. 6H, strain HX86 achieved an increased 3M1B titer by 1.2-fold, while a comparable IB titer, after expression of PK and PCK. Due to the slower growth rate of HX86 (Fig. 6G), the IB and 3M1B production per cell was significantly enhanced by 1.2-fold and 1.4-fold, respectively, relative to strain HX79 (Fig. 6I).

Among the engineered strains, the molar ratio of IB and 3M1B observed in strains HX86 and HX77 differed significantly from its corresponding control strain (Additional file 1: Fig. S2). The observed redistribution of end-products, IB and 3M1B, is consistent with what was claimed previously by Cheah et al. [41] that heterologous expression of PCK caused a redistribution of aldehyde production (isobutyraldehyde and isovaleraldehyde) in *Synechococcus*. Interestingly, both strains HX86 and HX77 had PCK additionally expressed, compared to its corresponding control strain. Therefore, PCK expression may directly or indirectly rearrange the metabolic flux of the branched-chain amino acid biosynthesis pathway, and further affect the molar ratio of IB and 3M1B. In conclusion, expression of the five selected target genes of central carbon metabolism showed positive effects on IB and 3M1B production. Co-expressing two of the selected targets successfully enhanced IB or 3M1B titer and production per cell (Fig. 6C–F, H–I). Not only being specifically valuable for evaluating and improving IB and 3M1B production derived from the 2-keto acid pathway, the identified gene targets may potentially be applied in metabolically engineering *Synechocystis* to produce various pyruvate-derived compounds.

## Conclusions

This study explicitly explored the 2-keto acid pathway for photosynthetic isobutanol (IB) and 3-methyl-1-butanol (3M1B) production in *Synechocystis* sp. PCC 6803. Enhanced IB and 3M1B production was observed after increasing *kivd*^*S286T*^ copy number, indicating α-ketoisovalerate decarboxylase as a rate-limiting enzyme. Moreover, overexpression of five gene targets of the central carbon metabolism effectively increased IB and 3M1B production, which are potential targets for overexpression to enhance any pyruvate-derived bioproduction. In the end, the maximum cumulative IB and 3M1B titers, 1247 mg L^−1^ and 389 mg L^−1^, obtained by strains HX29 and HX42, respectively, represent the currently highest reported.

### Supplementary Information

**Additional file 1.** Additional figures and tables.

## Data Availability

The datasets used and/or analyzed during the current study are available from the corresponding author on reasonable request.

## Abbreviations

3M1B: 3-Methyl-1-butanol
3PGA: 3-Phosphoglycerate
Adh: Alcohol dehydrogenase (EC:1.1.1.2)
AHAS: Acetohydroxy-acid synthase (EC 2.2.1.6)
AlsS: Acetolactate synthase (EC 2.2.1.6)
CBB: Calvin–Benson–Bassham
Cm^R^: Chloramphenicol resistance
CRISPR: Clustered regularly interspaced short palindromic repeats
Ddh: D-Lactate dehydrogenase (EC 1.1.1.28)
DHAP: Dihydroxyacetone phosphate
E4P: Erythrose-4-phosphate
*E. coli*: *Escherichia coli*
Em^R^: Erythromycin resistance
Eno: Enolase (EC 4.2.1.11)
F6P: Fructose-6-phosphate
FBA: Aldolase (EC 4.1.2.13)
FBP: Fructose-1,6-bisphosphate
G3P: Glyceraldehyde 3-phosphate
Gap1: Glyceraldehyde 3-phosphate dehydrogenase (EC:1.2.1.12)
Gpm: 2,3-Bisphosphoglycerate-independent phosphoglycerate mutase (EC:5.4.2.12)
IB: Isobutanol
IlvC: Acetohydroxy-acid isomeroreductase (EC:1.1.1.86)
IlvD: Dihydroxy-acid dehydratase (EC:4.2.1.9)
Kivd: α-Ketoisovalerate decarboxylase (EC:4.1.1.72)
Km^R^: Kanamycin resistance
LB: Lysogeny broth
LeuA: 2-Isopropylmalate synthase (EC:2.3.3.13)
LeuB: 3-Isopropylmalate dehydrogenase (EC:1.1.1.85)
LeuCD: 3-Isopropylmalate dehydratase (EC 4.2.1.33)
NSII: Neutral site II
OAA: Oxaloacetate
OD_750_: Optical density
OPP: Oxidative pentose phosphate
PCK: Phosphoenolpyruvate carboxykinase (EC:4.1.1.32)
PEP: Phosphoenolpyruvate
PDH: Pyruvate dehydrogenase E1 component (EC:1.2.4.1)
PEPc: Phosphoenolpyruvate carboxylase (EC:4.1.1.31)
Pgk: Phosphoglycerate kinase (EC:2.7.2.3)
PK: Pyruvate kinase (EC:2.7.1.40)
R5P: Ribose-5-phosphate
Ru5P: Ribulose-5-phosphate
Rubisco: Ribulose-1,5-bisphosphate carboxylase/oxygenase (EC:4.1.1.39)
RuBP: Ribulose-1,5-bisphosphate
S7P: Sedoheptulose-7-phosphate
SBP: Sedoheptulose-1,7-bisphosphate
Slr1192.^OP^: Codon-optimized alcohol dehydrogenase (EC:1.1.1.2)
Sp^R^: Spectinomycin resistance
*Synechocystis*: *Synechocystis* sp. PCC 6803
TCA cycle: Tricarboxylic acid cycle
TK: Transketolase (EC:2.2.1.1)
TPI: Triosephosphate isomerase (EC:5.3.1.1)
Xu5P: Xylulose-5-phosphate
