# Supplementary material for: Sustainable production of photosynthetic isobutanol and 3-methyl-1-butanol in the cyanobacterium Synechocystis sp. PCC 6803
Source: Biotechnol Biofuels Bioprod. 2023 Sep 9;16:134. doi: 10.1186/s13068-023-02385-1 (PMC10492371; doi:10.1186/s13068-023-02385-1)
Supplement: Supplementary file 1 — Additional file 1. Additional figures and tables. [file 13068_2023_2385_MOESM1_ESM.pdf]

Sustainable production of photosynthetic isobutanol and 3-methyl-1-butanol in the cyanobacterium *Synechocystis* PCC 6803

Hao Xie, Jarl Kjellström, Peter Lindblad*

hao.xie@kemi.uu.se
kjellstrom.jarl@yahoo.se
peter.lindblad@kemi.uu.se

Microbial Chemistry, Department of Chemistry-Ångström Laboratory, Uppsala University, Box 523, SE-75120 Uppsala, Sweden

<u>Additional file 1</u>

Figures S1 - S2
Tables S1 - S3

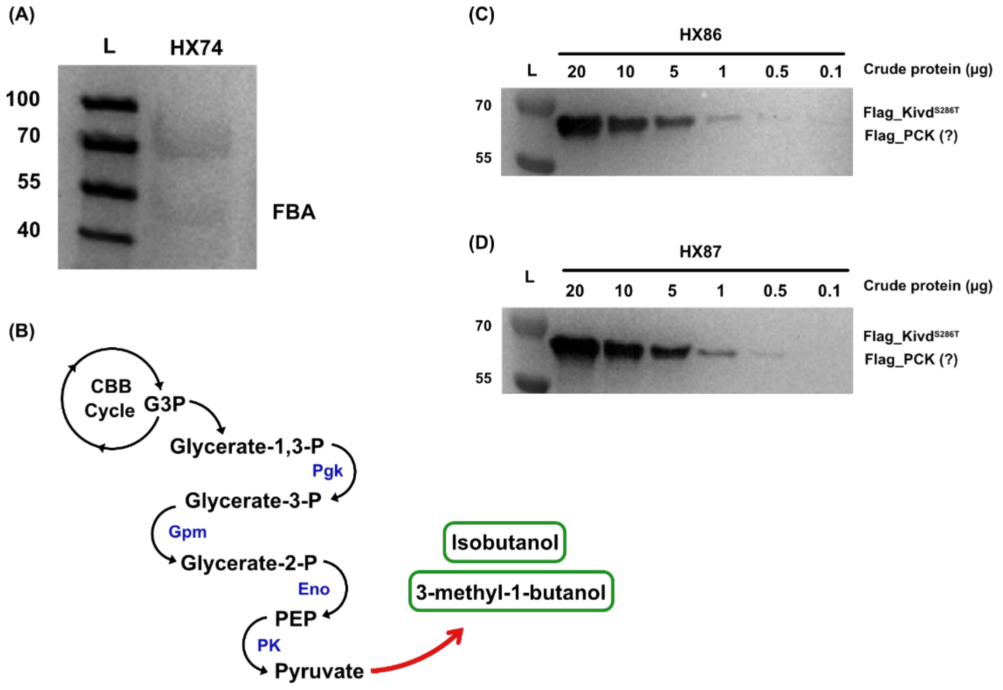

Figure S1 Western-immunoblot analysis of selected strains and central carbon metabolic pathway with selected reactions. (A) Western-immunoblot analysis of His-tagged FBA expression in *Synechocystis* PCC 6803 strain HX74. L, ladder (in kDa). 162 μg of total soluble proteins were loaded for detection. Protein size: FBA, 39 kDa. (B) Simplified pathway between metabolites G3P and pyruvate. G3P is an intermediate metabolite of Calvin-Benson-Bassham (CBB) cycle, and pyruvate is a starting metabolite for isobutanol and 3-methyl-1-butanol biosynthesis through 2-keto acid pathway. Endogenous pathways are written in black; heterologous pathways are written in red. Abbreviations of enzymes: Pgk, phosphoglycerate kinase; Gpm, 2,3-bisphosphoglycerate-indepndent phosphoglycerate mutase; Eno, enolase; PK, pyruvate kinase. Abbreviations of intermediates: G3P, glyceraldehyde-3-phosphate; glycerate-1,3-P, glycerate-1,3-bisphosphate; glycerate-3-P, glycerate-3-phosphate; glycerate-2-P, glycerate-2-phosphate; PEP, phosphoenolpyruvate. (C) Western-immunoblot analysis of Flag-tagged Kivd^{S286T} and PCK expression in engineered *Synechocystis* strain HX 86. Crude proteins were loaded in a series of dilutions (20 μg, 10 μg, 5 μg, 1 μg, 0.5 μg, and 0.1 μg). L, ladder (in kDa). Protein size: Kivd^{S286T}, 61 kDa; PCK, 60 kDa. (D) Western-immunoblot analysis of Flag-tagged Kivd^{S286T} and PCK expression in engineered *Synechocystis* strain HX 87. Crude proteins were loaded in a series of dilutions (20 μg, 10 μg, 5 μg, 1 μg, 0.5 μg, and 0.1 μg). L, ladder (in kDa). Protein size: Kivd^{S286T}, 61 kDa; PCK, 60 kDa.

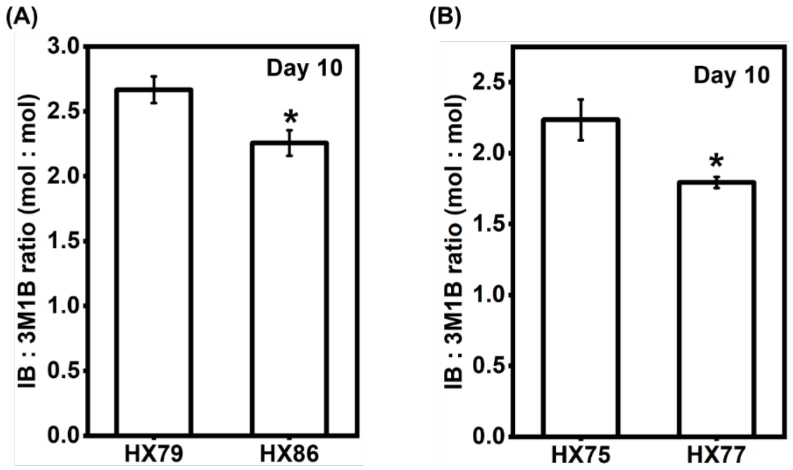

Figure S2 Molar ratio of isobutanol (IB) and 3-methyl-1-butanol (3M1B) in engineered *Synechocystis* PCC 6803 strains HX75, HX77, HX79 and HX86. (A) IB:3M1B molar ration of strains HX79 and HX86 on day 10. (B) IB:3M1B molar ration of strains HX75 and HX77 on day 10. Results are the mean of three biological replicates, each with three technical replicates. Error bars represent standard deviation. Asterisk represents significant difference between different strains (one-way ANOVA, *p < 0.05).

Table S1 Plasmids used in this study. Expressed genes in bold

Plasmid	Relevant characteristics[a]	Reference
P1	pDdh-(P*trc*BCD-***slr1192***^{OP}-***sll0065***-T)-Cm^{R}	This study
P2	pDdh-(P*trc*BCD-***slr1192***^{OP}-***slr2088***-***sll0065***-T)-Cm^{R}	This study
P3	pSlr0168-(P*trc*BCD-***sll1363***-***slr0452***-T)-Sp^{R}	This study
P4	pSlr0168-(P*psbA2*-***sll1363***-***slr0452***-T)-Sp^{R}	This study
P5	pEEK2-(P*trc*BCD-***kivd***^{S286T}-T)-Km^{R}	(Miao et al., 2018)
P6	pDdh-(P*trc*BCD-***kivd***^{S286T}-T)-Cm^{R}	(Xie and Lindblad, 2022)
P7	pSll1564-(P*trc*BCD-***kivd***^{S286T}-T)-Sp^{R}	(Xie and Lindblad, 2022)
P8	pSlr0186-(P*trc*BCD-***kivd***^{S286T}-T)-Sp^{R}	(Xie and Lindblad, 2022)
P9	pPEPc-(P*trc*BCD-***kivd***^{S286T}-T)-Sp^{R}	(Xie and Lindblad, 2022)
P10	pSlr1934-(P*trc*BCD-***kivd***^{S286T}-T)-Sp^{R}	(Xie and Lindblad, 2022)
P11	pSll1721-(P*trc*BCD-***kivd***^{S286T}-T)-Sp^{R}	(Xie and Lindblad, 2022)
P12	pSlr0168-(P*trc*BCD-***kivd***^{S286T}-T)-Em^{R}	This study
P13	pSlr0168-(P*psbA2*-T)-Em^{R}	This study
P14	pDdh-(P*trc*BCD-***slr1192***^{OP}-***alsS***-T)-Cm^{R}	(Xie and Lindblad, 2022)
P15	pSlr0168-(P*trc*BCD-***kivd***^{S286T}-T)-Sp^{R}	(Xie and Lindblad, 2022)
P16	pSlr0168-(P*trc*BCD-***kivd***^{S286T}-T)-Sp^{R}	(Xie and Lindblad, 2022)
P17	pSlr0168-(P*trc*BCD-***ilvC***-***ilvD***-T)-Sp^{R}	(Xie and Lindblad, 2022)
P18	pNSII-(P*nrsB*-T)-Em^{R}	This study
P19	pNSII-(P*trc*BCD-***fbaA***-***tktA***-T)-Em^{R}	This study
P20	pNSII-(P*trc*BCD-***pckA***-***tpiA***-T)-Em^{R}	This study
P21	pNSII-(P*trc*BCD-***pyk1***-***pckA***-T)-Em^{R}	This study

[a] NSII, neutral site II, a region between *slr2030* and *slr2031*; Km^{R}, kanamycin resistance cassette; Sp^{R}, spectinomycin resistance cassette; Cm^{R}: chloramphenicol resistance cassette; Em^{R}, erythromycin resistance cassette; T, Terminator BBa_B0015. The difference between P15 and P16 is different tags linked to *kivd*^{S286T}: P15, His tag; P16, Flag tag.

Table S2 Oligonucleotides used in this study

Primer name	Oligonucleotides sequence
**A. Primers for PCR amplification of homologous recombination regions**	
ddh_US_BglII_F	TATAAGATCTAAACCACTGGGCCAGTAGTTC
ddh_US_Ter_EcoRI_R	TATAGAATTCAAAAAAAGGATCTCAAGAAGATCCTTTGATTTTGACGATTATGGGAAGTAGTTTAG
ddh_DS_BamHI_F	TATAGGATCCGGTTAGAAAATATCAATGTTAAC
ddh_DS_SalI_R	TATAGTCGACCGGACTATTTGGTAGAACATAAAATC
slr0168_US_BglII_F	TATAAGATCTAATGTGGAACGGGGCCTAGACAC
slr0168_US_Ter_EcoRI_R	TATAGAATTCAAAAAAAGGATCTCAAGAAGATCCTTTGATTTTAGATTAATTCAACAGTAATATTTC
slr0168_DS_BamHI_F	TATAGGATCCCTCAGGGGCATTATCGGAGCAAG
slr0168_DS_XhoI_R	TATACTCGAGGATCGCCAAAGATGTTGGCCGTCGGG
NSII_US_BglII_F	TATAAGATCTTTGTTAAAAAATCATCGGGCCGGTC
NSII_US_Ter_EcoRI_R	TATAGAATTCAAAAAAAGGATCTCAAGAAGATCCTTTGATTTTCCAAATGGTTTGAGCAAAGTTATTC
NSII_DS_BamHI_F	TATAGGATCCGTGGTCATTCTCAAGGAGTTGGTGGCTAAGTTG
NSII_DS_SalI_R	TATAGTCGACCGGCCCGCCACAATAGGGGAG
**B. Primers for *Synechocystis* colony PCR to verify genomic integration**	
ddh_UUS_F	CAGCCGTACCACTTCTTCTAC
slr0168_UUS_F	ATGTGGAGTTTGTGGGTATTG
NSII_UUS_F	GCTTTCTCCCCTCTACGTTTCCATCCATG
slr1192^{OP}_SR	AGTTACTAATACCCCATTCGTTG
sll1363_SR	TTTACTCCCGGAATATAAACCC
kivd^{S286T}_SR	TTGGCATTACCCACCCATTTC
EmR_SR	CGGTACCAATTTCATAAACGGTGTC
fbaA_SR	TACCGTAACCGTTTTCGGCAG
pckA_SR	GTTCTTGCGGGGTCAAACCATTG
pyk1_SR	ATAAATTTCACGGGGTTAATCAG
**C. Primers for PCR with *Synechocystis* genomic DNA to examine fully segregation**	
ddh_SF	AGTCCATGTACCATCCTATTCTC
ddh_SR	GCTCTTCCTCTTCTTCGTAAAC
slr0168_SF	CGGCAATGATCCAGAGAATG
slr0168_SR	CTATCTGCCAAAGCTGCTTC
NSII_SF	ACCCCGGCAATTTTGTTGAA
NSII_SR	AGTAACTTACGACGGGTGGG

**D. Primers for *Synechocystis* colony PCR to verify self-replicating plasmid conjugation**	
VF2	TGCCACCTGACGTCTAAGAA
kivd^{S286T}_SR	TTGGCATTACCCACCCATTTC
**E. Primers for PCR amplification of genes**	
kivd^{S286T}_BglII_F	TATAAGATCTATGTACACCGTGGGAGATTAC
SpeI_PstI_R	CTGCAGCGGCCGCTACTAG
slr1192^{OP}_BglII_F	TATAAGATCTATTAAAGCCTACGCCGCCTTG
sll1363_BglII_F	TATAAGATCTCAAAACTTGATTTTACAACATTTGC
sll1363_His_XbaI_F	TATATCTAGAATGCATCATCACCATCACCACGGTAGCGGAAGTGGATCTCAAAAC
XbaI_F	TATATCTAGATAGTGGAGGTACTAGAATG
fbaA_BglII_F	TATAAGATCTGCCTTAGTGCCCATGC
tktA_RBS_Strep_XbaI_F	TATATCTAGATAGTGGAGGTACTAGAATGTGGAGTCATCCTCAGTTCGAGAAGGGTAGCGGAAGTGGATCTGTTGTTGCTAC
pckA_BglII_F	TATAAGATCTCGCGTTAACAATGGTTTGACCCC
pckA_Spe_Pst_R	TATACTGCAGCGGCCGCTACTAGTTTACAGTTTCGGACCAGCCGCTAC
tpiA_RBS_Flag_F	TATATCTAGATAGTGGAGGTACTAGAATGGACTACAAGGATGACGATGACAAGGGTAGCGGAAGTGGATCTCGACATCCTTTAGTGATGGGTAAC
tpiA_Spe_Pst_R	TATACTGCAGCGGCCGCTACTAGTTTAAGCCTGTTTAGCCGCTTC
pyk1_BglII_F	TATAAGATCTCCCGCCCTGATTAACC
pckA_RBS_Flag_XbaI_F	TATATCTAGATAGTGGAGGTACTAGAATGGACTACAAGGATGACGATGACAAGGGTAGCGGAAGTGGATCTCGCGTTAACAATGGTTTGACCCC

Table S3 Sequences of all genes used in this study

Gene	Nucleotide sequence (5’-3’)
kivd^{S286T}	ATGTACACCGTGGGAGATTACTTACTGGACCGCTTACATGAACTGGGTATTGAA<br>GAAATTTTTGGCGTGCCCGGAGATTACAACTTACAATTTTTAGATCAAATTATTA<br>GTCATAAAGACATGAAATGGGTGGGTAATGCCAACGAACTGAATGCCTCCTATA<br>TGGCTGATGGGTACGCCCGTACCAAAAAAGCCGCTGCCTTTTTGACCACTTTT<br>GGCGTGGGTGAACTGTCCGCCGTTAATGGCTTGGCTGGTAGTTATGCCGAAAA<br>CTTACCCGTGGTTGAAATTGTGGGGTCCCCCACCTCCAAAGTTCAAAACGAAG<br>GAAAATTTGTGCATCACACCTTGGCCGATGGCGACTTTAAACATTTTATGAAAAT<br>GCACGAACCCGTGACTGCTGCCCGGACCTTGTTAACTGCCGAAAATGCTACCG<br>TGGAAATTGATCGTGTTCTGAGTGCTCTGTTGAAAGAACGGAAACCCGTGTACA<br>TTAACTTGCCCGTGGACGTTGCTGCCGCTAAAGCCGAAAAACCCTCCTTACCC<br>CTGAAAAAAGAAAATTCCACCAGTAACACTTCTGATCAAGAAATTTTAAACAAAA<br>TTCAGGAATCCCTGAAAAACGCCAAAAAACCCATTGTTATTACTGGCCACGAAA<br>TTATTAGTTTTGGTTTGGAAAAAACCGTGACTCAATTTATTTCCAAAACCAAATT<br>GCCCATTACCACTCTGAATTTTGGGAAATCCAGTGTGGATGAAGCCCTGCCCT<br>CCTTTTTGGGCATTTATAATGGCACCTTGAGTGAACCCAACCTGAAAGAATTTG<br>TTGAAAGCGCTGATTTTATTTTGATGCTGGGGGTGAAATTGACCGACACCTCTA<br>CTGGAGCCTTTACCCATCACCTGAACGAAAACAAAATGATTAGTCTGAACATTG<br>ATGAAGGTAAAATCTTTAACGAACGGATTCAAAACTTTGATTTTGAATCCTTGAT<br>TTCCAGTTTACTGGACTTATCTGAAATTGAATACAAAGGGAAATACATTGATAAA<br>AAACAGGAAGACTTTGTGCCCAGCAATGCTTTGTTATCTCAAGATCGCCTGTGG<br>CAGGCCGTTGAAAATTTGACTCAAAGTAACGAAACCATTGTGGCTGAACAGGG<br>CACCTCCTTTTTCGGAGCCAGCTCTATTTTTCTGAAAAGCAAATCTCATTTTATT<br>GGACAACCCTTATGGGGGAGTATTGGATATACCTTTCCCGCCGCTTTGGGCAG<br>CCAGATTGCCGATAAAGAATCTCGCCACCTGTTGTTTATTGGGGACGGCTCCTT<br>GCAATTGACCGTTCAGGAATTAGGCCTGGCCATTCGCGAAAAAATTAACCCCAT<br>TTGTTTTATTATTAACAACGATGGCTACACCGTGGAACGTGAAATTCATGGTCC<br>CAATCAATCCTATAACGACATTCCCATGTGGAATTACTCCAAATTGCCCGAAAG<br>TTTTGGTGCCACTGAAGATCGGGTGGTTTCTAAAATTGTGCGCACCGAAAATGA<br>ATTTGTGAGCGTTATGAAAGAAGCCCAGGCTGACCCCAACCGTATGTACTGGA<br>TTGAATTGATTCTGGCCAAAGAAGGTGCCCCCAAAGTGCTGAAGAAAATGGGA<br>AAACTGTTTGCTGAACAGAATAAAAGCTAA
alsS	ATGTTGACCAAAGCCACTAAAGAACAAAAATCCTTAGTGAAAAACCGCGGTGCC<br>GAATTGGTGGTTGATTGTTTAGTGGAACAGGGGGTGACCCATGTTTTTGGGATT<br>CCCGGAGCCAAAATTGATGCTGTGTTTGACGCCTTGCAAGATAAAGGTCCCGA<br>AATTATTGTGGCCCGCCACGAACAAAACGCTGCTTTTATGGCCCAGGCTGTGG<br>GGCGTTTAACCGGCAAACCCGGTGTGGTTCTGGTTACTAGCGGCCCCGGTGC<br>TTCTAATTTAGCCACCGGGTTGTTAACCGCCAACACTGAAGGAGATCCCGTGG<br>TTGCCCTGGCTGGCAATGTGATTCGGGCCGACCGCTTGAAACGTACCCATCAA<br>TCCCTGGATAACGCCGCTTTGTTTCAGCCCATTACTAAATATAGTGTGGAAGTT<br>CAAGATGTGAAAAATATTCCCGAAGCTGTTACCAACGCCTTTCGTATTGCCTCC<br>GCTGGGCAAGCCGGAGCCGCTTTTGTGAGTTTTCCCCAGGATGTGGTTAACGA<br>AGTTACCAACACTAAAAACGTGCGGGCCGTTGCCGCTCCCAAATTGGGACCCG<br>CCGCTGATGACGCCATTAGCGCCGCTATTGCTAAAATTCAAACCGCCAAATTGC<br>CCGTGGTTCTGGTGGGGATGAAAGGCGGTCGCCCCGAAGCCATTAAAGCTGT<br>GCGTAAACTGTTGAAAAAAGTGCAATTACCCTTTGTTGAAACCTATCAGGCCGC<br>TGGTACTTTGAGTCGGGATTTAGAAGACCAATACTTTGGCCGTATTGGTTTATTT<br>CGGAATCAACCCGGAGACTTACTGTTGGAACAGGCCGATGTGGTTCTGACCAT<br>TGGCTATGACCCCATTGAATACGATCCCAAATTTTGGAACATTAACGGCGACCG<br>CACTATTATTCATTTGGATGAAATTATTGCTGATATTGACCACGCCTATCAACCC

	GACTTAGAACTGATTGGTGATATTCCCAGTACCATTAATCATATTGAACACGATG<br>CTGTGAAAGTTGAATTTGCCGAACGTGAACAGAAAATTCTGAGCGATTTGAAAC<br>AATACATGCATGAAGGGGAACAGGTGCCCGCTGACTGGAAATCTGATCGGGCC<br>CACCCCTTGGAAATTGTGAAAGAATTACGCAATGCCGTTGATGACCATGTGACC<br>GTTACTTGCGATATTGGCTCCCACGCCATTTGGATGTCCCGGTATTTTCGCAGT<br>TACGAACCCTTGACCTTAATGATTAGCAACGGCATGCAAACTTTAGGCGTGGCT<br>TTGCCCTGGGCTATTGGAGCTTCTTTAGTGAAACCCGGTGAAAAAGTGGTTTCC<br>GTTAGTGGTGATGGGGGATTTCTGTTTAGTGCTATGGAATTGGAAACCGCTGT<br>GCGGTTAAAAGCCCCCATTGTGCATATTGTTTGGAATGACTCCACCTATGATAT<br>GGTGGCCTTTCAACAGTTGAAAAAATACAATCGCACCTCCGCCGTGGATTTTGG<br>CAACATTGATATTGTTAAATACGCTGAATCCTTTGGGGCCACCGGATTACGTGT<br>GGAATCTCCCGACCAATTAGCTGATGTTCTGCGGCAGGGGATGAATGCCGAAG<br>GACCCGTGATTATTGATGTGCCCGTTGATTACAGCGACAATATTAACTTAGCCT<br>CTGATAAACTGCCCAAAGAATTTGGCGAACTGATGAAAACCAAAGCCTTGTAA
sll0065	ATGGAATTTTATCCCAATGGTCATCGGCGCTCCCCCTCCTTGCCTCCCATGAAA<br>CATACCTTGTCCGTGTTAGTGGAAGATGAAGCCGGCGTGTTGACCCGCATTGC<br>CGGTTTATTTGCCCGTCGGGGGTTTAATATTGAATCCTTGGCCGTGGGGAGTG<br>CCGAACAAGGCGATGTGAGTCGTATTACTATGGTGGTGCCCGGCGATGAAAAT<br>ACCATTGAACAATTAACCAAACAATTGTACAAATTAGTGAATGTGATTAAAGTGC<br>AAGATATTACCGAAACCCCCTGTGTGGAACGTGAATTGATGTTAGTGAAAGTGT<br>CCGCCAATGCCCCCAATCGGGCCGAAGTGATTGAATTGGCCCAAGTGTTTCGC<br>GCCCGTATTGTGGATATTAGTGAAGATACCGTGACCATTGAAGTGGTGGGCGA<br>TCCCGGTAAAATGGTGGCCATTTTGCAAATGTTAGCCAAATTTGGCATTAAAGA<br>AGTGGCCCGGACCGGTAAAATTGCCTTAGTGCGCGAATCCGGCGTGAACACC<br>GAATATTTGAAATCCTTAGAAAGTAAATTTTAA
slr2088	ATGGTGGCCTCCATTCCCAATCCCAAAACCAATTTTCTGAAAACCGTGCCCTCC<br>CAACGTCAAACCGGTGCCTACATTTTGATGGATAGTTTGAAACGGCATGGCGT<br>GAAACATATTTTTGGTTATCCCGGCGGTGCCATTTTACCCATTTATGATGAATTG<br>TATCGCTTTGAAGCCGCCGGTGAAATTGAACATATTTTGGTGCGTCATGAACAA<br>GGTGCCTCCCATGCCGCCGATGGTTATGCCCGTGCCACCGGTAAAGTGGGCG<br>TGTGTTTTGGGACCAGTGGTCCCGGCGCCACCAATTTAGTGACCGGCATTGCC<br>AATGCCCATTTGGATTCCGTGCCTATGGTGGTGATTACCGGTCAAGTGGGGCG<br>GGCCATGATTGGTAGTGATGCCTTTCAAGAAATTGATATTTTTGGGATTACCTTA<br>CCCATTGTGAAACATTCCTATGTGGTGCGGAGTGCCGCCGATATGGCTCGTAT<br>TGTGACCGAAGCCTTTCATTTAGCCTCCACCGGCCGGCCCGGTCCCGTGTTAA<br>TTGATATTCCCAAAGATGTGGGCTTGGAAGAATGTGAATATATTCCCTTAGATC<br>CCGGCGATGTGAATTTGCCCGGCTATCGTCCCACCGTGAAAGGTAATCCCCGG<br>CAAATTAATGCCGCCTTACAATTGTTAGAACAAGCCCGGAATCCCTTGTTATAT<br>GTGGGCGGCGGCGCCATTGCTGCTAATGCTCATGCCCAAGTGCAAGAATTTGC<br>CGAACGCTTTCAATTACCCGTGACCACCACCTTGATGGGTATTGGGGCCTTTG<br>ATGAAAATCATCCCTTATCCGTGGGGATGTTGGGCATGCATGGCACCGCCTAT<br>GCCAATTTTGCCGTGAGTGAATGTGATTTGTTAATTGCCGTGGGTGCCCGGTTT<br>GATGATCGCGTGACCGGCAAATTGGATGAATTTGCCAGTCGCGCCAAAGTGAT<br>TCATATTGATATTGATCCCGCCGAAGTGGGCAAAAATCGTGCCCCCGATGTGC<br>CCATTGTGGGTGATGTGCGCCATGTGTTAGAACAATTGTTACAACGGGCCCGC<br>GAATTGGATTATCCCACCCATCCCCATACCACCCAAGCCTGGTTAAATCGTATT<br>GATCATTGGCGGACCGATTATCCCTTGCAAGTGCCCCATTATGAAGATACCATT<br>GCTCCCCAAGAAGTGGTGCATGAAATTGGCCGTCAAGCCCCCGATGCCTATTA<br>TACCACCGATGTGGGTCAACATCAAATGTGGGCCGCCCAATTTTTAAATAATGG<br>TCCCCGTCGGTGGATTTCCAGTGCCGGCTTAGGCACTATGGGCTTTGGTTTGC<br>CCGCCGCTATGGGCGCCAAAGTGGGTGTGGGGGATGAAGCCGTGATTTGTAT<br>TTCCGGTGATGCCAGTTTTCAAATGAATTTGCAAGAATTAGGGACCTTGGCCCA

	ATACGATATTCAAGTGAAAACCATTATTTTGAACAACGGGTGGCAAGGCATGGT<br>GCGCCAATGGCAACAAACCTTTTATGAAGAACGTTATTCCGCCAGTAATATGAG<br>TCAAGGGATGCCCGATATTAACTTGTTGTGTGAAGCCTATGGTATTAAAGGGAT<br>TACCGTGCGCAAACGTGAAGATTTAGCCCCCGCCATTGCCGAAATGTTGGCCC<br>ATAATGGCCCCGTGGTGATGGATGTGGTGGTGAAAAAAGATGAAAACTGTTAC<br>CCCATGATTGCCCCCGGTATGTCCAATGCCCAAATGTTGGGTTTACCCGAAGT<br>GCCCGTGCGCGATAATGGCCCCCGTATGGTGGAATGTAACCATTGTCAAACCC<br>AAAACTTTATTACCCATCGCTTTTGTAGTGGCTGTGGTGCCAAATTGTAA
ilvC	ATGGCCAACTATTTTAACACCCTGAACTTACGCCAACAACTGGCTCAACTGGGG<br>AAATGCCGCTTTATGGGCCGTGACGAATTTGCTGACGGAGCCAGTTATTTGCA<br>AGGCAAAAAAGTGGTTATTGTGGGCTGTGGTGCCCAAGGGCTGAATCAGGGAT<br>TGAACATGCGCGATTCCGGTCTGGACATTAGTTATGCCTTGCGTAAAGAAGCTA<br>TTGCCGAAAAACGTGCCTCCTGGCGGAAAGCTACCGAAAACGGCTTTAAAGTG<br>GGTACTTACGAAGAATTAATTCCCCAAGCCGATTTAGTTATTAATCTGACCCCC<br>GATAAACAACATAGCGACGTGGTTCGGACTGTGCAGCCCTTAATGAAAGATGG<br>GGCCGCTCTGGGATATTCTCACGGCTTTAATATTGTGGAAGTTGGGGAACAAAT<br>TCGCAAAGACATTACCGTGGTTATGGTGGCCCCCAAATGCCCCGGCACTGAAG<br>TTCGCGAAGAATACAAACGTGGGTTTGGAGTGCCCACCTTGATTGCCGTTCAT<br>CCCGAAAATGATCCCAAAGGCGAGGGTATGGCTATTGCCAAAGCCTGGGCCG<br>CTGCCACCGGCGGTCACCGTGCTGGCGTGCTGGAATCCAGTTTTGTGGCTGA<br>AGTTAAATCCGATTTGATGGGTGAACAAACCATTTTGTGTGGCATGTTACAGGC<br>CGGGAGTTTGCTGTGCTTTGATAAATTAGTGGAAGAAGGAACCGACCCCGCCT<br>ATGCTGAAAAATTGATTCAATTTGGGTGGGAAACCATTACTGAAGCCCTGAAAC<br>AAGGCGGCATTACCTTAATGATGGATCGTCTGTCCAACCCCGCCAAACTGCGG<br>GCCTACGCTCTGAGTGAACAATTGAAAGAAATTATGGCCCCCTTATTTCAGAAA<br>CACATGGATGACATTATTTCCGGAGAATTTAGCTCTGGCATGATGGCCGATTGG<br>GCTAATGATGACAAAAAACTGTTGACCTGGCGCGAAGAAACCGGCAAAACTGC<br>CTTTGAAACTGCTCCCCAATATGAAGGGAAAATTGGAGAACAGGAATACTTTGA<br>TAAGGGTGTGTTGATGATTGCTATGGTGAAAGCTGGGGTTGAATTAGCCTTTGA<br>AACTATGGTGGATAGTGGTATTATTGAAGAAAGCGCCTATTACGAATCTTTGCA<br>TGAATTGCCCTTAATTGCCAATACCATTGCTCGGAAACGCTTATATGAAATGAA<br>CGTGGTTATTAGCGATACTGCCGAATATGGCAATTACTTGTTTTCTTACGCTTGT<br>GTGCCCTTACTGAAACCCTTTATGGCCGAATTGCAACCCGGCGACTTAGGCAA<br>AGCCATTCCCGAAGGTGCTGTGGATAATGGGCAGTTACGGGACGTTAACGAAG<br>CCATTCGCTCCCACGCTATTGAACAGGTTGGGAAAAAATTGCGTGGATACATGA<br>CCGATATGAAACGGATTGCTGTGGCTGGTTAA
sll1363	ATGCAAAACTTGATTTTACAACATTTGCAAGATCGTATTAGTCGGCCCGCCTTT<br>GCCATGATTAAAAGTTTACAATCCACCATTCAAAGTCAACAGGGTGATTCCTATA<br>TGGCCCGTATGTATTATGATCAAGATGCCAATTTGGATTTGTTAGCCGGGAAAA<br>CCGTGGCCATTATTGGCTATGGTAGTCAAGGGCATGCCCATGCCTTGAATTTAA<br>AAGATTCCGGCGTGAATGTGGTGGTGGGTTTATATTCCGGGAGTAAATCCGTG<br>GCCAAAGCCGAAGGGGCCGGCTTGAAAGTGTTATCCGTGGCCGAAGCCGCCA<br>AAGCCGCCGATTTGATTATGATTTTGTTACCCGATGAAGTGCAAAAAACCGTGT<br>ATGAAGCCGAAATTGCCCCCAATTTAGTGGCCGGTAATGTGTTGTTGTTTGCCC<br>ACGGCTTTAACATTAACTTTGCCCAAATTGTGCCTCCCGCCGATGTGGATGTGG<br>TGATGGCTGCTCCCAAAGGTCCCGGCCATTTGGTGCGTCGGACCTATGAACAA<br>GGCCAAGGTGTGCCCGCCTTATTTGCCGTGTATCAAGATGCCAGTGGGCAAGC<br>CCGCGATTATGCTATGGCCTATGCCAAAGGTATTGGCGGCACCCGCGCCGGC<br>ATTTTGGAAACCACCTTTCGCGAAGAAACCGAAACCGATTTGTTTGGTGAACAA<br>GTGGTGTTATGTGGTGGCTTGACCGCCTTGATTAAAGCCGGCTTTGATACCTTA<br>GTGGAAGCCGGTTATCAACCCGAATTGGCCTACTTTGAATGTTTGCATGAAGTG<br>AAATTGATTGTGGATTTAATTGTGGAAGGTGGGTTGGCCAAAATGCGCGATAGT<br>ATTTCCAATACCGCCGAATATGGCGATTTAACCCGCGGTCCCCGTATTGTGACC

	GAAGAAACCAAAGCCGAAATGCGCCAAATTTTGGATGAAATTCAATCCGGCCAA<br>TTTGCCCGTGAATTTGTGTTAGAAAATCAAGCCGGGAAACCCGGTTTTACCGCC<br>ATGCGTCGGCGCGAAAGTGAAGAATTGATTGAAGAAGTGGGTAAAGATTTGCG<br>TGCCATGTTTTCCTGGTTAAAAGATCGGTAA
ilvD	ATGCCCAAATATCGTAGTGCCACCACTACCCACGGCCGTAATATGGCTGGTGC<br>CCGCGCTTTGTGGCGTGCTACTGGAATGACCGACGCTGATTTTGGTAAACCCA<br>TTATTGCCGTGGTTAACTCCTTTACCCAATTTGTGCCCGGCCATGTGCACCTGC<br>GGGATTTGGGAAAATTAGTGGCCGAACAGATTGAAGCCGCTGGCGGTGTTGCT<br>AAAGAATTTAATACCATTGCCGTGGATGACGGGATTGCTATGGGACATGGGGG<br>AATGCTGTATAGTTTGCCCAGCCGTGAATTAATTGCCGATTCTGTTGAATACAT<br>GGTGAATGCTCACTGCGCCGACGCTATGGTGTGCATTAGCAACTGTGATAAAA<br>TTACTCCCGGCATGTTGATGGCCTCTTTGCGCTTAAATATTCCCGTTATTTTTGT<br>GTCCGGCGGTCCTATGGAAGCCGGTAAAACCAAATTGAGTGACCAAATTATTAA<br>ACTGGACTTGGTGGATGCCATGATTCAGGGGGCTGATCCCAAAGTTTCCGACA<br>GTCAAAGCGATCAGGTGGAACGCAGTGCCTGTCCCACCTGCGGCTCTTGTTCC<br>GGCATGTTTACTGCCAATAGCATGAACTGTTTGACCGAAGCCTTGGGGCTGTC<br>TCAACCCGGTAATGGCTCCTTGTTAGCCACTCATGCTGATCGCAAACAGTTGTT<br>TTTAAACGCCGGCAAACGCATTGTGGAATTGACCAAACGTTACTACGAACAAAA<br>CGATGAAAGTGCCTTACCCCGTAACATTGCTAGCAAAGCCGCTTTTGAAAATGC<br>CATGACCCTGGATATTGCTATGGGCGGCTCCACCAACACCGTGTTGCACCTGT<br>TGGCCGCTGCCCAAGAAGCCGAAATTGATTTTACTATGATGAGTGACATTGATA<br>AACTGAGCCGTAAAGTTCCCCAATTGTGTAAAGTGGCCCCCTCCACCCAGAAA<br>TATCACATGGAAGATGTTCACCGGGCCGGCGGTGTGATTGGGATTTTAGGAGA<br>ACTGGACCGGGCCGGGTTACTGAATCGCGATGTTAAAAACGTGCTGGGCTTGA<br>CTTTACCCCAAACCTTAGAACAGTACGATGTTATGCTGACCCAAGATGACGCCG<br>TGAAAAATATGTTTCGTGCTGGACCCGCTGGTATTCGCACTACCCAAGCCTTTA<br>GTCAGGACTGCCGGTGGGATACCTTAGATGACGATCGTGCCAACGGCTGTATT<br>CGGTCTCTGGAACATGCTTATTCCAAAGATGGGGGATTGGCCGTGTTATACGG<br>CAATTTTGCTGAAAACGGTTGCATTGTTAAAACTGCCGGCGTGGACGATTCTAT<br>TTTGAAATTTACCGGTCCCGCCAAAGTTTATGAATCCCAAGACGATGCCGTGGA<br>AGCTATTTTAGGCGGTAAAGTGGTTGCCGGTGATGTGGTTGTGATTCGGTATG<br>AAGGGCCCAAAGGGGGACCCGGAATGCAAGAAATGTTGTACCCCACCTCTTTT<br>CTGAAAAGTATGGGCTTGGGTAAAGCCTGTGCTCTGATTACTGATGGACGTTTT<br>TCCGGCGGCACCTCCGGATTATCCATTGGACACGTGAGCCCCGAAGCTGCCA<br>GTGGGGGAAGCATTGGGTTAATTGAAGACGGCGATTTGATTGCCATTGACATT<br>CCCAATCGGGGCATTCAATTGCAGGTGTCCGATGCCGAATTAGCTGCTCGTCG<br>GGAAGCTCAAGATGCTCGCGGTGATAAAGCCTGGACTCCCAAAAATCGTGAAC<br>GGCAAGTTAGTTTTGCCTTGCGCGCCTACGCTAGTTTAGCCACCAGCGCTGAC<br>AAAGGTGCCGTGCGTGATAAATCCAAATTAGGCGGTTAA
slr0452	ATGTCCAATAATCCCCGCAGTCAAGTGATTACCCAAGGCACCCAACGCAGTCC<br>CAATCGTGCCATGTTACGGGCCGTGGGTTTTGGGGATGATGATTTTACCAAAC<br>CCATTGTGGGTATTGCCAATGGGTATTCCACCATTACCCCCTGTAATATGGGCA<br>TTAATGATTTGGCCTTGCGGGCTGAAGCTGGTTTACGTACCGCCGGTGCTATG<br>CCCCAATTGTTTGGGACCATTACCATTTCCGATGGCATTAGTATGGGGACCGAA<br>GGCATGAAATATTCCTTGGTGAGTCGTGAAGTGATTGCCGATAGTATTGAAACC<br>GTGTGTAATGGGCAACGGATGGATGGCGTGTTAGCCATTGGCGGTTGTGATAA<br>AAATATGCCCGGTGCCATGATTGCTATGGCCCGTTTGAATATTCCCTCCATTTT<br>TGTGTATGGGGGCACCATTAAACCCGGTCATTATGCCGGTGAAGATTTAACCG<br>TGGTGTCCGCCTTTGAAGCCGTGGGTCAATATAGTGCCGGGAAAATTGATGAA<br>GAAACCTTGTATGGCATTGAACGGAATGCTTGTCCCGGCGCTGGTAGTTGTGG<br>TGGGATGTTTACCGCCAATACCATGTCCAGTGCCTTTGAAGCTATGGGCATGTC<br>CTTACCCTATTCCTCCACTATGGCCGCCGTGGATGGCGAAAAAGCCGATTCCA

	CCGAAGAAAGTGCCAAAGTGTTGGTGGAAGCCATCAAAAAACAAATTTTGCCCA<br>GTCAAATTTTAACCCGGAAAGCCTTTGAAAATGCCATTGCCGTGATTATGGCCG<br>TGGGCGGTAGTACCAATGCCGTGTTACATTTGTTAGCCATTGCCAATACCATTG<br>GGGTGCCCTTGTCCTTAGATGATTTTGAAACCATTCGCCATAAAGTGCCCGTGT<br>TGTGTGATTTAAAACCCTCCGGCAAATATGTGACCACCAATTTGCATGCCGCCG<br>GGGGCATTCCCCAAGTGATGAAAATTTTGTTAGTGAACGGGATTTTGCATGGC<br>GATGCCTTAACCATTACCGGCCAAACCATTGCCGAAGTGTTAGCCGATATTCCC<br>GATCAACCTCCCGCCGGCCAAGATGTGATTCATAGTTGGGATGATCCCGTGTA<br>TCAAGAAGGGCATTTGGCCGTGTTAAAGGGTAATTTGGCCACCGAAGGGTCCG<br>TGGCCAAAATTAGTGGTGTGAAAAAACCCGTGATTACCGGGCCCGCCAAAGTG<br>TTTGAATCCGAAGAAGATTGTTTGGAAGCCATTTTAGCCGGCAAAATTCAAGCC<br>GGTGATGTGGTGGTGGTGCGGTATGAAGGCCCCAAAGGTGGGCCCGGTATGC<br>GCGAAATGTTAGCTCCCACCTCCGCTATTATTGGTGCTGGTTTGGGTGATAGTG<br>TGGGGTTAATTACCGATGGCCGCTTTTCCGGCGGCACCTATGGCTTGGTGGTG<br>GGTCATGTGGCTCCCGAAGCCTATGTGGGCGGCGCCATTGCCTTGGTGCAAG<br>AAGGCGATCAAATTACCATTGATGCCGGTAAACGTTTGTTACAATTAAACATTAG<br>TGAAGAAGAATTGGCTCAACGGCGCGCCCAATGGACCCCTCCCCAACCCCGG<br>TATCCCCGCGGCATTTTGGCCAAATATGCCAAATTAGTGTCCAGTTCCAGTTTG<br>GGTGCCGTGACCGATATTGATTTGTTTTAA
slr1192^{OP}	ATGATTAAAGCCTACGCCGCCTTGGAAGCCAATGGAAAATTACAGCCCTTTGAA<br>TACGATCCCGGTGCCCTGGGTGCTAACGAAGTTGAAATTGAAGTGCAATATTGT<br>GGCGTGTGTCATAGTGATTTGTCCATGATTAACAACGAATGGGGTATTAGTAAC<br>TATCCCTTAGTGCCCGGTCATGAAGTGGTGGGGACCGTGGCTGCTATGGGCG<br>AAGGTGTGAATCATGTGGAAGTGGGGGATTTAGTGGGGTTGGGCTGGCATAGT<br>GGCTATTGTATGACCTGTCATAGTTGTTTGTCCGGTTATCATAACTTGTGTGCC<br>ACCGCCGAATCCACCATTGTGGGGCATTATGGCGGTTTTGGCGATCGGGTGC<br>GCGCCAAAGGGGTGAGTGTGGTGAAATTGCCCAAAGGCATTGATTTGGCCTCC<br>GCCGGTCCCTTATTTTGTGGTGGCATTACCGTGTTTAGTCCTATGGTGGAATTG<br>TCCTTAAAACCCACCGCCAAAGTGGCCGTGATTGGCATTGGTGGGTTAGGTCA<br>TTTGGCCGTGCAATTTTTGCGTGCCTGGGGCTGTGAAGTGACCGCCTTTACCA<br>GTTCCGCCCGGAAACAAACCGAAGTGTTGGAATTAGGGGCCCATCATATTTTA<br>GATAGTACCAATCCCGAAGCCATTGCCTCCGCCGAAGGCAAATTTGATTACATT<br>ATTAGTACCGTGAACTTGAAATTGGATTGGAACTTGTACATTTCCACCTTGGCT<br>CCCCAAGGTCATTTTCATTTTGTGGGCGTGGTGTTGGAACCCTTGGATTTAAAT<br>TTGTTTCCCTTGTTGATGGGTCAACGCTCCGTGAGTGCTTCCCCCGTGGGGAG<br>TCCCGCCACCATTGCCACCATGTTAGATTTTGCCGTGCGTCATGATATTAAACC<br>CGTGGTGGAACAATTTTCCTTTGATCAAATTAACGAAGCCATTGCTCATTTGGA<br>AAGTGGTAAAGCCCATTATCGGGTGGTGCTGTCCCATTCTAAAAACTAA
fbaA	ATGGCCTTAGTGCCCATGCGCCTGCTGCTGGACCACGCTGCCGAAAACGGTTA<br>CGGTATTCCCGCCTTTAATGTGAATAATATGGAACAGATTATTTCTATTATGCAA<br>GCCGCTGATGAAACCGACTCCCCCGTGATTTTGCAAGCCAGTCGGGGCGCCC<br>GGTCCTATGCCGGAGAAAATTTTCTGCGTCATTTAGTGCTGGGTGCTGTTGAAA<br>CCTACCCCCACATTCCCATTGCCATGCATCAAGATCATGGCAATTCCCCCGCTA<br>CCTGTTATTCCGCCATTCGGAACGGATTTACCTCCGTGATGATGGATGGCAGC<br>TTAGAAGCTGACGCCAAAACCCCCGCTAGTTTTGAATACAATGTGAACGTTACT<br>GCCGAAGTGGTTAAAGTGGCTCATTCTGTTGGTGCCTCCGTGGAAGGGGAACT<br>GGGATGCTTGGGCTCTTTAGAAACCGGGCAAGGAGAAGCCGAAGATGGTCAT<br>GGCTTTGAAGGGAAACTGGATCACAGTCAATTGTTAACTGACCCCGAAGAAGC<br>CGTGGAATTTGTTAATAAAACCCAGGTTGACGCTTTGGCCGTGGCTATTGGCAC<br>CAGTCATGGCGCCTATAAATTTACCCGCAAACCCACTGGAGAAGTGTTAGCCAT<br>TAGCCGTATTGAAGAAATTCACCGGCTGTTGCCCAATACCCATCTGGTTATGCA<br>CGGGTCCAGTAGCGTGCCCCAAGAATGGATTGATATGATTAACGAATTTGGCG

	GTGCCATTCCCGAAACCTACGGCGTGCCCGTTGAAGAAATTCAGAAAGGAATT AAATCCGGCGTGCGTAAAGTTAATATTGATACCGACAACCGCTTGGCCATTACT GCCGCTTTTCGTGAAGCCGCTGCCAAAGATCCCAAAAATTTTGACCCCCGGCA TTTTCTGAAACCCAGCATTAAATACATGAAACAAGTGTGTGCCGATCGCTACCA ACAGTTTTGGACCGCCGGCAACGCTTCCAAAATTAAACAGTTGACTTTAGATGA CTATGCTGCCAAATACGCCAAAGGGGAACTGACTGCCACTTCTCGCACTTCTG TTGCTGTTTAA
tktA	ATGGTTGTTGCTACCCAAAGTCTGGACGAACTGTCTATTAACGCCATTCGGTTT CTGGCTGTTGACGCCATTGAAAAAGCTAAAAGCGGCCATCCCGGCTTGCCTAT GGGCGCCGCTCCTATGGCCTTTACCCTGTGGAACAAATTTATGAAATTTAATCC CAAAAACCCCAAATGGTTTAACCGGGATCGCTTTGTGTTGTCCGCCGGTCATG GCTCCATGTTACAATATGCCTTGTTATACCTGTTGGGGTATGATTCCGTGACCA TTGAAGACATTAAACAATTTCGCCAGTGGGAATCCAGTACCCCCGGCCACCCC GAAAATTTTCTGACTGCCGGGGTGGAAGTTACCACTGGACCCTTGGGGCAGGG AATTGCCAATGGCGTGGGTCTGGCCTTGGCTGAAGCCCATTTAGCCGCTACCT ATAACAAACCCGATGCCACCATTGTGGACCACTATACTTACGTTATTTTGGGGG ATGGATGTAATATGGAAGGGATTAGCGGAGAAGCCGCTTCTATTGCCGGCCAT TGGGGCTTGGGTAAATTGATTGCCCTGTACGATGACAACCACATTAGCATTGAT GGTTCTACCGACGTGGCCTTTACTGAAGATGTTTCCAAACGTTTTGAAGCCTAT GGCTGGCATGTGCTGCACGTTGAAGATGGTAATACCGACTTGGCCGCTATTGC TAAAGCCATTGAAGAAGCTAAAGCCGTGACCGATAAACCCTCCATGATTAAAGT TACCACTATTATTGGGTACGGAGCCCCCAATAAAAGTGATACCGCTGGCATTCA TGGTGCCGCTTTAGGCACCGACGAAGTGGCCGCTACTCGGAAAAACCTGGGTT GGGATTATGCCCCCTTTGAAGTGCCCCAAGAAGTTTTAGACTACACCCGCAAA GCCATTGAACGTGGTGCCAGTTATGAAGCTGAATGGAATCAAGCCTTTGCCCA GTACAAAACCAAATATCCCACTGAAGCCGCTGCCTTTGAACGCCAATTAAGCG GGGCCCTGCCCGAAGGATGGGATAAAACCTTAGCCTCTTTTACTCCCGACCAG AAAGGCCTGGCCACCCGTAAATACAGTGAAGAATGCTTGAATGCTTTAGCCCC CGTGCTGCCCGAATTGATTGGCGGTTCCGCCGATTTGACCCATAGTAACTTAA CTGAACTGCACTGTAGCGGCGACTTTCAAAAAGGTGCCTATCAGAATCGGAAC GTGCATTTTGGTGTTCGCGAACACGCTATGGGCGCTATTTGCAATGGAATTGC CTTACATGGCTCCGGTTTACTGCCCTTTGGCGCCACCTTTTTGATTTTTACTGAT TACATGCGTGCTGCCATTCGGTTGAGCGCCTTATCTGAAGCTGGGGTGATTTG GGTTATGACCCATGATAGCATTGGGCAAGGAGAAGACGGCCCCACCCACCAG CCCATTGAAGTGCTGGCCTCTTTGCGTGCTATTCCCAATTTAACCGTTATTCGG CCCGCCGATGGCAACGAAACCTCCGGTGCTTACAAAGTGGCTATTGCCAAAGC TAAAGAAAATGCCCCCACCTTGTTATCCCTGACTCGTCAAGCCGTGCCCAACTT GCCCGGCACCTCCATTGATGCCGTTGCTAAAGGAGCCTATACTATTGTGGACA GCGAAGGGGTTCCCGAATTAATTCTGATTGGCACCGGTTCTGAAGTGCAATTAT GTGTTGCTGCCGCTGAAAAACTGGCCGCTCAGGGTAAAAAAGTGCGGGTGGTT TCCATGCCCAGTTGGGAATTGTTTGAAACCCAAGATGCCGCTTACAAAGAATCC GTGTTGCCCAAAGCCGTTACCAAACGCTTAAGTGTGGAAGCCGCTACTAATTTT GGTTGGCATAAATATGTGGGGACCGAAGGAGATACTGTTAGCATTGAAACCTTT GGGGCCTCTGCCCCCGGCGGCGTGTGCCTGGAAAAATTTGGATTTAGTGTTGA TAATGTGTTAGCCAAAGCCAAAACCTTGTTGTCCTAA
pckA	CGCGTTAACAATGGTTTGACCCCGCAAGAACTCGAGGCTTATGGTATCAGTGA CGTACATGATATCGTTTACAACCCAAGCTACGACCTGCTGTATCAGGAAGAGCT CGATCCGAGCCTGACAGGTTATGAGCGCGGGGTGTTAACTAATCTGGGTGCC GTTGCCGTCGATACCGGGATCTTCACCGGTCGTTCACCAAAAGATAAGTATATC GTCCGTGACGATACCACTCGCGATACTTTCTGGTGGGCAGACAAAGGCAAAGG TAAGAACGACAACAAACCTCTCTCTCCGGAAACCTGGCAGCATCTGAAAGGCC TGGTGACCAGGCAGCTTTCCGGCAAACGTCTGTTCGTTGTCGACGCTTTCTGT

	GGTGCGAACCCGGATACTCGTCTTTCCGTCCGTTTCATCACCGAAGTGGCCTG<br>GCAGGCGCATTTTGTCAAAAACATGTTTATTCGCCCGAGCGATGAAGAACTGG<br>CAGGTTTCAAACCAGACTTTATCGTTATGAACGGCGCGAAGTGCACTAACCCG<br>CAGTGGAAAGAACAGGGTCTCAACTCCGAAAACTTCGTGGCGTTTAACCTGAC<br>CGAGCGCATGCAGCTGATTGGCGGCACCTGGTACGGCGGCGAAATGAAGAAA<br>GGGATGTTCTCGATGATGAACTACCTGCTGCCGCTGAAAGGTATCGCTTCTAT<br>GCACTGCTCCGCCAACGTTGGTGAGAAAGGCGATGTTGCGGTGTTCTTCGGCC<br>TTTCCGGCACCGGTAAAACCACCCTTTCCACCGACCCGAAACGTCGCCTGATT<br>GGCGATGACGAACACGGCTGGGACGATGACGGCGTGTTTAACTTCGAAGGCG<br>GCTGCTACGCAAAAACTATCAAGCTGTCGAAAGAAGCGGAACCTGAAATCTAC<br>AACGCTATCCGTCGTGATGCGTTGCTGGAAAACGTCACCGTGCGTGAAGATGG<br>CACTATCGACTTTGATGATGGTTCAAAAACCGAGAACACCCGCGTTTCTTATCC<br>GATCTATCACATCGATAACATTGTTAAGCCGGTTTCCAAAGCGGGCCACGCGA<br>CTAAGGTTATCTTCCTGACTGCTGATGCTTTCGGCGTGTTGCCGCCGGTTTCTC<br>GCCTGACTGCCGATCAAACCCAGTATCACTTCCTCTCTGGCTTCACCGCCAAA<br>CTGGCCGGTACTGAGCGTGGCATCACCGAACCGACGCCAACCTTCTCCGCTT<br>GCTTCGGCGCGGCATTCCTGTCGCTGCACCCGACTCAGTACGCAGAAGTGCT<br>GGTGAAACGTATGCAGGCGGCGGGCGCGCAGGCTTATCTGGTTAACACTGGC<br>TGGAACGGCACTGGCAAACGTATCTCGATTAAAGATACCCGCGCCATTATCGA<br>CGCCATCCTCAACGGTTCGCTGGATAATGCAGAAACCTTCACTCTGCCGATGTT<br>TAACCTGGCGATCCCAACCGAACTGCCGGGCGTAGACACGAAGATTCTCGATC<br>CGCGTAACACCTACGCTTCTCCGGAACAGTGGCAGGAAAAAGCCGAAACCCTG<br>GCGAAACTGTTTATCGACAACTTCGATAAATACACCGACACCCCTGCGGGTGC<br>CGCGCTGGTAGCGGCTGGTCCGAAACTGTAA
tpiA	ATGCGACATCCTTTAGTGATGGGTAACTGGAAACTGAACGGCAGCCGCCACAT<br>GGTTCACGAGCTGGTTTCTAACCTGCGTAAAGAGCTGGCAGGTGTTGCTGGCT<br>GTGCGGTTGCAATCGCACCACCGGAAATGTATATCGATATGGCGAAGCGCGAA<br>GCTGAAGGCAGCCACATCATGCTGGGTGCGCAAAACGTGGACCTGAACCTGT<br>CCGGCGCATTCACCGGTGAAACCTCTGCTGCTATGCTGAAAGACATCGGCGCA<br>CAGTACATCATCATCGGTCACTCTGAACGTCGTACTTACCACAAAGAATCTGAC<br>GAACTGATCGCGAAAAAATTCGCGGTGCTGAAAGAGCAGGGCCTGACTCCGGT<br>TCTGTGCATCGGTGAAACCGAAGCTGAAAATGAAGCGGGCAAAACTGAAGAAG<br>TTTGCGCACGTCAGATCGACGCGGTACTGAAAACTCAGGGTGCTGCGGCATTC<br>GAAGGTGCGGTTATCGCTTACGAACCTGTATGGGCAATCGGTACTGGCAAATC<br>TGCAACTCCGGCTCAGGCACAGGCTGTTCACAAATTCATCCGTGACCACATCG<br>CTAAAGTTGACGCTAACATCGCTGAACAAGTGATCATTCAGTACGGCGGCTCT<br>GTAAACGCGTCTAACGCTGCAGAACTGTTTGCTCAGCCGGATATCGACGGCGC<br>GCTGGTTGGTGGTGCTTCTCTGAAAGCTGACGCCTTCGCAGTAATCGTTAAAG<br>CTGCAGAAGCGGCTAAACAGGCTTAA
pyk1	ATGCCCGCCCTGATTAACCCCGTGAAATTTATGCGCCCCTTATCTCATCGGACT<br>AAAATTGTTGCTACTATTGGACCCGCCTCCTCCTCCGTGGAAGTTATTCGTCAA<br>ATGGTGGATGCCGGGATGAATGTTGCTCGGTTAAACTTTAGTCATGGCTCCTAT<br>GAAGATCACGCCACTATGGTGCGCTTGTTACGTTCCGTTGAACAGGAAATGGA<br>TACCCCCATTACTCTGTTGCAAGACTTGCAGGGGCCCAAAATTCGCATTGGACA<br>ACTGCCCGGCGGTGAAAAACAGTTGCGTGAAGGCGAAAAAGTGAGTTTAGTGC<br>CCGTTGAAATTGGTGATCGCCATCCCGGCGCCGTGGGTATTGACTACCCCCAC<br>TTGGCCACCGAAGCTAAAGTGGGCGAACGTATTTTACTGGATGACGGTTTGTTA<br>GAAATGAAAGTGGTTTCTATTCAAGATCCCGAAGTGATTTGTGAAGTGGTTACC<br>GGGGGAATTTTAAAATCCCGTAAAGGGGTGAATCTGCCCGGACTGGTTTTGAC<br>TTTACCCAGTATGACCACTAAAGATAAACAAGACCTGGAATTTGGCTTGAGCCA<br>GGGTATTGATTGGGTGTCCTTGAGTTTTGTTCGTAAAGGGGAAGACATTCATAC<br>CTTAAAACAATTTCTGGCCGAACGGGGACACCCCGATTTACCCGTGATTGCCA

	AAATTGAAAAACCCCAGGCTATTGACAATTTGGAAGAAATTGTGGCCGTTTCCA ACGGCATTATGGTGGCTCGCGGGGATTTAGGAGTGGAAGTTAATCCCGAAAAA GTGCCCCGGCTGCAAAAAGAAATTATTCGTCGGTGCAACGTGCGCGCCATTCC CGTTATTACCGCTACTCAAATGCTGGATAGTATGATTCAGAATAGCCGGCCCAC CCGCGCCGAAGCTAGTGATGTGGCCAACGCTATTTTGGATGGGACTGACGCC GTGATGTTATCCGGCGAAAGTGCTGTTGGTCAATATCCCGTGAAAAGCGTTCA GATGTTGCGGAAAATTGCCGAAGAAACCGAAGTGGGCCTGCATTTGGTTAACA ATCCTCCCATTGAAAACACCGAAACTCACGCCTTGAGTGAAGCCTTGGTGGTTA TTGATGGCATTTTAGACCTGAAATACATTGTGACCTTTACCACTAGCGGTTTTAC TTCTCTGTTGGCCTCCAATCAACGCCCCTCCGTGCCCGTTATTGCCTTTACCCC CTCTGAAAAAGTGTACCATTCCTTGAACCTGGTTTGGGGTATTATTCCCTTTCTG ATTAACGAAGAATTTGATACCTTTGAAGACTTGATTCAACAGGCCGAAGTGTTA CTGCGTGATCGGAAAATGGTGGAAAAAGGGGACCAATTGTTAATTATGGCTGG CATTCCCACCAAAATTCCCCGTGGCACCAACTTTCTGAAAATTCATCGTATTAG C

References

1. Miao R, Xie H, Ho FM, Lindblad P. Protein engineering of alpha-ketoisovalerate decarboxylase for improved isobutanol production in *Synechocystis* PCC 6803. *Metab Eng*. 2018;47:42-48.
2. Xie H, Lindblad P. Expressing 2-keto acid pathway enzymes significantly increases photosynthetic isobutanol production. *Microb Cell Fact*. 2022;21(1):17.
